# Mycobacterial heat shock protein 65 mediated metabolic shift in decidualization of human endometrial stromal cells

**DOI:** 10.1038/s41598-017-04024-w

**Published:** 2017-06-21

**Authors:** Elavarasan Subramani, Arun Prabhu Rameshbabu, Manivannan Jothiramajayam, Bhuvaneshwaran Subramanian, Debangana Chakravorty, Gunja Bose, Mamata Joshi, Chaitali Datta Ray, Indrani Lodh, Ratna Chattopadhyay, Sudipto Saha, Anita Mukherjee, Santanu Dhara, Baidyanath Chakravarty, Koel Chaudhury

**Affiliations:** 1 School of Medical Science and Technology, Indian Institute of Technology Kharagpur, Kharagpur, 721302 West Bengal India; 2 Cell Biology and Genetic Toxicology Laboratory, Centre of Advanced study, Department of Botany, University of Calcutta, Kolkata, 700019 West Bengal India; 3 Bioinformatics Centre, Bose Institute, Kolkata, 700054 West Bengal India; 4 Institute of Reproductive Medicine, Kolkata, 700020 West Bengal India; 5 National Facility for High-field NMR, Tata Institute of Fundamental Research, Mumbai, 400005 Maharashtra India; 6 Department of Gynaecology and Obstetrics, Institute of Post-Graduate Medical Education and Research (IPGMER) and SSKM Hospital, Kolkata, 700020 West Bengal India

## Abstract

Successful implantation is dependent on the appropriate decidualization of endometrial stromal cells for the establishment of pregnancy in women. Mycobacterial heat shock protein 65 (HSP65) is involved in pathogenesis of the genital tuberculosis (GTB), one of the common causes of infertility in emerging countries. Though implantation failure appears to be the major cause, understanding the status of decidualizaiton process in women diagnosed with GTB has not been thoroughly addressed. We, therefore, explored the effect of HSP65 protein on the endometrial cell metabolism during *in vitro* decidualization. In order to identify the cellular metabolism of decidual cells with and without HSP65 treatment, proton NMR based characterization of metabolites extracted from cells and culture media were performed. In presence of HSP65, significant reduction in the decidual phenotype of endometrial stromal cells and prolactin expression is suggestive of impairment in decidualization. The intracellular and extracellular metabolic changes in HSP65 treated endometrial stromal cells produced a distinct pattern, reflecting the interaction between the protein and cellular metabolism. HSP65 mediated dysregulation in cellular metabolism is associated with poor decidualization. Besides enriching the present knowledge on metabolic changes underlying stromal cells decidualization, these findings assist in identifying potential molecular causes for decidualization failure in GTB women.

## Introduction

Genital tuberculosis (GTB) is common in infertile women with increasing prevalence of 5% worldwide^1, 2^. Importantly, GTB is associated with implantation failure, even in its dormant form^2, 3^. The 65 kDa heat shock protein (HSP65), secreted by *Mycobacterium tuberculosis* during intracellular survival, is one of the major immunologically active antigens and considered to be a diagnostic marker of GTB^4^. This protein plays an important role in the pathogenesis of tuberculosis^4, 5^ and may contribute to the implantation failure in GTB women.

The process of successful embryo implantation into the endometrium occurs in three stages including apposition, adhesion and invasion^6^. Blastocyst apposition and adhesion steps ensure that the trophoblast cells adhere to the receptive endometrium and form a strong embryo-endometrial linkage. During the invasion process, human endometrial stromal cells (hESC) undergo the process of decidualization to mediate harmonized interaction between a receptive endometrium and a functional blastocyst^7^. Decidualization is a process involving morphological and biochemical/metabolic changes of the hESC through the action of ovarian steroids, 17β-estradiol and progesterone^7, 8^. Endometrial decidualization provides energy for the trophoblast invasion and functional placenta formation^9, 10^. Transformation of fibroblast hESC into decidual-type cells occurs during the mid-late secretory phase and produces prolactin and insulin-like growth factor-binding protein 1 (IGFBP-1), which are well established decidualization markers^10^. Accumulating evidences suggest that endometrial and pregnancy complications are frequently associated with abnormal decidualization due to impairment in proliferation and differentiation of stromal cells^11, 12^.

Global metabolite profiling (metabolomics) provides a comprehensive understanding of the metabolic differences in cells/tissues^13–15^. Proton nuclear magnetic resonance spectroscopy (1H NMR) is being commonly used for the quantitative assessment of low molecular weight metabolites and functional read-out of cellular phenotypic responses to disease and/or any chemical treatment^14, 16^. In an earlier study, we used 1H NMR metabonomics and could successfully characterize the metabolic profile of the endometrium obtained from women with GTB and healthy controls^17^. This study revealed the possible association of dysregulated energy metabolism and amino acid biosynthesis metabolites with repeated implantation failure in dormant GTB women^4^.

In view of these findings, it is likely that the synchronized cross-talk between the embryo and the endometrium is affected due to compromised apposition and adhesion in dormant GTB women. Furthermore, in another study, we observed aberrant decidualization of hESC upon treatment with Mycobacterial HSP65 *in vitro*. This was attributed to reduced cytokine mediated signal transducer and activator of transcription 3 (STAT3) signaling^4^. These findings motivated us to focus on Mycobacterial HSP65 mediated metabolic reprogramming in hESC decidualized cells in an *in vitro* model. To address this, 1H NMR metabonomics was used to examine intracellular and secretory metabolic alterations of hESC under HSP65 stimuli. Additionally, various computational tools were applied to explore the biochemical mechanism of decidualization process under normal and altered conditions. This study also reveals, for the first time, the metabolic flux of hESC during the process of decidualization. The findings hold promise for providing mechanistic insights into the regulation of hESC metabolism associated with decidualization failure in women with GTB.

## Results

### HSP65 induced aberrant endometrial decidualization

**Figure 1:**
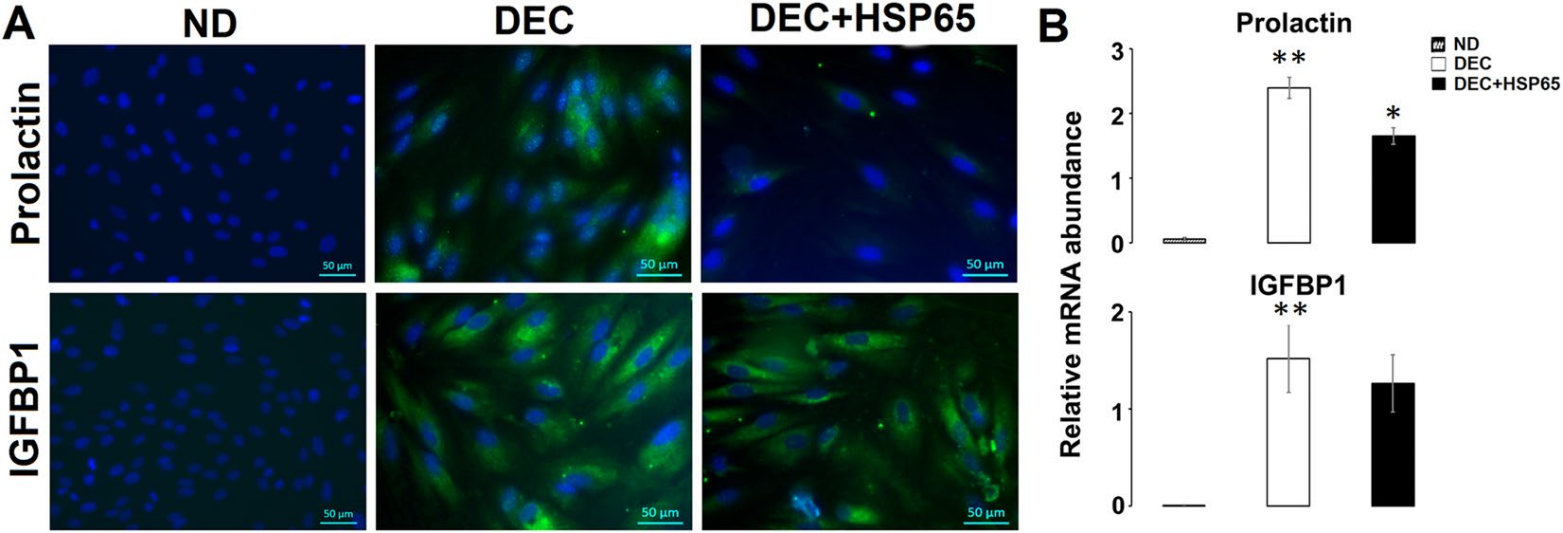
HSP65 alters prolactin mediated endometrial stromal cells (hESC) decidualization. Protein (**A**) and mRNA (**B**) levels of prolactin and IGFBP1, decidualization markers, in non-decidualized hESC (ND), decidualized hESC (DEC) and HSP65-treated decidualized hESC (DEC + HSP65). ND shows no expression of decidualization markers. A significant down regulation in the expression of prolactin in HSP65 treated decidualized cells. Data are represented as Mean ± SEM. *P < 0.05, **P < 0.0001.

On treatment with 8-bromoadenosine 3′, 5′-cyclic monophosphate (cAMP) and medroxyprogesterone acetate (MPA) for decidualization, changes in hESC from fibroblast-like to enlarged polygonal cell morphology, a typical characteristic of decidualized cells, was observed. These morphological changes were further confirmed by transcript abundance pattern and estimation of protein expression. The treatment was found to increase mRNA and protein levels of prolactin and IGFBP1, which are well established decidualization markers (Fig. 1A and B). Next, on pretreatment with HSP65, most of the hESC remained as fibroblastic cells, with significant reduction in prolactin expression. However, IGFBP1 levels were found to be comparable (Fig. 1A and B).

### Discrimination between decidualized hESC with and without HSP65 treatment and non-decidualized cells

**Figure 2:**
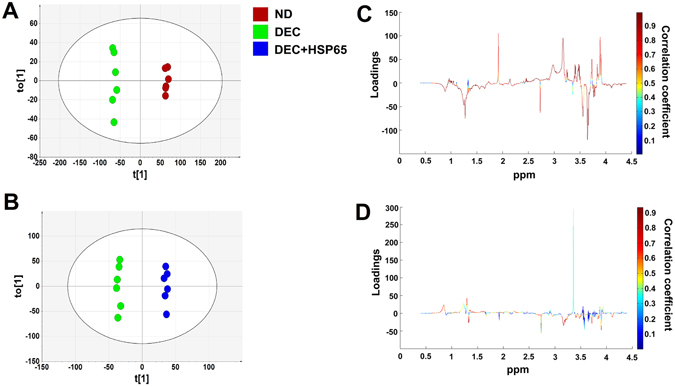
Chemometric analysis of decidualized hESC treated with and without HSP65 and non-decidualized cells. Two dimensional scatter score plot of OPLS-DA of ND vs DEC (**A**), OPLS-DA of DEC vs DEC + HSP65 (**B**). The coefficient loading plots of OPLS-DA models (ND vs DEC (**C**); DEC vs DEC + HSP65 (**D**)) is represented as color maps (MATLAB R2009a, The MathWorks, Inc., USA). The color bar is indicating the modulus of correlation.

Chemometric analysis was used to classify the groups based on differences in metabolites expression of hESC during decidualization with and without HSP65 treatment. For visualizing the inherent clustering of groups, NMR spectra of all three groups were subjected to principal component analysis (PCA). The PCA scatter plot indicated separation of non-decidualized hESC (ND) from decidualized cells (DEC); however, no class separation between HSP65 treated (DEC + HSP65) and untreated decidualized cells was observed (Supplementary Figure S1A; R2X = 85.4%, Q2 = 0.766; Goodness of fit (R2), Goodness of prediction (Q2)). Improved separation was achieved by partial least squares discriminant analysis (PLS-DA), the supervised classification model (Supplementary Figure S1B; R2X = 87.4%, R2Y = 0.977 and Q2 = 0.925). Further, orthogonal PLS-DA (OPLS-DA) model was applied to optimize classification between the groups by removing unrelated variables of the class. We also used this model to extract the significant metabolites discriminating the groups. Improved separation and prediction of classes could be observed using this model [ND vs DEC (Fig. 2A; R2X = 79.6%, R2Y = 0.997, Q2 = 0.985 and analysis of variance testing of cross validated predictive residuals (CV-ANOVA) score (p = 0.0077654); DEC vs DEC + HSP65 (Fig. 2B; R2X = 78.1%, R2Y = 0.997, Q2 = 0.825 and CV-ANOVA score (p = 6.82 × 10^−7^))]. Predictability and accuracy of the model were also validated by ROC analysis (area under ROC = 0.99). S-plot and variable important for projection (VIP) plot with threshold of >±0.60 and ≥1 respectively, could successfully identify significantly dysregulated metabolites responsible for effective discrimination between the groups (Fig. 2C-ND vs DEC; Fig. 2D-DEC vs DEC + HSP65).

One dimensional (1D) 1H NMR peaks of decidualized and non-decidualized hESC corresponding to specific metabolites were identified using two dimensional (2D) NMR experiments [Total Correlation Spectroscopy (TOCSY) and Correlation Spectroscopy (COSY)] and literature^18, 19^. Twenty seven metabolites could be identified in 1D NOESY NMR spectra (Supplementary Table S2). The representative NMR spectra of decidualized hESC with metabolites assignment are shown in Supplementary Figure S2A.

Based on the significant variables identified in the S-plot and VIP scores, univariate analysis was applied to the integral values of these metabolites. Lactate, lipid, 3-hydroxybutyrate, acetate, alanine, lysine, histidine, tyrosine, proline and threonine were found to be significantly higher in DEC as compared to ND (Table 1). A significant decrease in D-glucose, creatine, myoinositol, choline, glycerophosphocholine, glutamate, isoleucine and leucine was observed in DEC compared with ND (Table 1). Furthermore, we observed a significant increase in lipid, 3-hydroxybutyrate, acetate, glutamate, alanine, lysine and proline and decrease in D-glucose, lactate, myoinositol, choline, glycerophosphocholine and taurine in DEC + HSP65 as compared to DEC (Table 1).

**Table 1:** Fold change and p-value of intracellular and extracellular metabolites discriminating the groups.

Metabolites	ppm	Fold change			
		Cells		Media	
		DEC vs ND	DEC + HSP65 vs DEC	DEC vs ND	DEC + HSP65 vs DEC
D-Glucose (m)	3.81	0.645*	0.804**	0.742*	0.463**
Lactate (d)	1.32	1.278**	0.891**	1.505*	0.537**
Pyruvate (s)	2.46	—	—	—	—
Creatine (s)	3.02	0.362***	—	NA	NA
Myoinositol (dd)	3.51	0.412***	0.516*	—	0.673***
Lipid (m)	0.88	3.778***	1.302*	NA	NA
Choline (s)	3.19	0.301**	0.579*	0.594**	0.583**
Glycerophosphocholine (s)	3.2	0.481*	0.479**	NA	NA
3-hydroxybutyrate (d)	1.2	2.912***	1.312*	0.625*	—
Acetate (s)	1.92	1.479*	1.465**	—	1.215**
Succinate (s)	2.39	—	—	1.364*	0.638***
Citrate (d)	2.54	—	—	1.993**	0.31**
Formate (s)	8.44	—	—	NA	NA
Glycine (s)	3.54	—	—	—	—
Glutamine (m)	2.12	—	—	1.267**	—
Glutamate (m)	2.36	0.593***	1.214*	—	—
Alanine (d)	1.46	5.727***	1.662***	1.057*	1.138**
Lysine (m)	1.72	2.587**	1.328**	—	—
Isoleucine (t)	0.92	0.855**	—	1.373**	0.564***
Leucine (t)	0.94	0.733**	—	—	—
Valine (d)	0.97	—	—	—	—
Histidine (m)	3.98	1.773***	—	0.764*	—
Phenylalanine (d)	7.32	—	—	1.159**	0.869**
Tyrosine (m)	6.87	3.734**	—	—	—
Proline (m)	2.34	2.016**	1.429*	1.305*	0.64***
Threonine (m)	4.24	3.072***	—	1.914**	—
Taurine (t)	3.25	—	0.629**	—	0.842*
1-methyl histidine (s)	7.67	NA	NA	—	—
Oxypurinol (s)	8.32	NA	NA	—	—

*P < 0.05; **p < 0.01; ***p < 0.0001; - Non significant; NA- not identified.

### Secretome of hESC during *in-vitro* decidualization with and without HSP65 treatment

**Figure 3:**
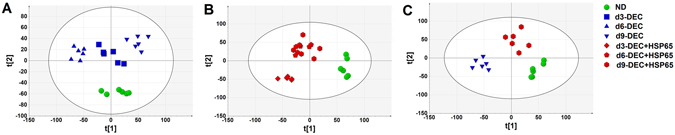
Multivariate analysis of extracelluar metabolites of hESC during *in vitro* decidualization. (**A**) Independent analysis of PLS-DA showed time dependent trend on comparing ND and d3, d6 and d9 culture media metabolomic profiles of DEC [R2X = 77.5%, R2Y = 0.990 and Q2 = 0.942]. (**B**) PLS-DA of ND and d3, d6 and d9 metabolomic profiles of DEC + HSP65 [R2X = 74.2%, R2Y = 0.978 and Q2 = 0.866] showed noticeable overlaps between d6 and d9 metabolomic profiles of DEC + HSP65. (**C**) PLS-DS revealed discrimination between metabolic profiles of ND, d9-DEC and d9-DEC + HSP65 [R2X = 83.6%, R2Y = 0.973 and Q2 = 0.827].

**Figure 4:**
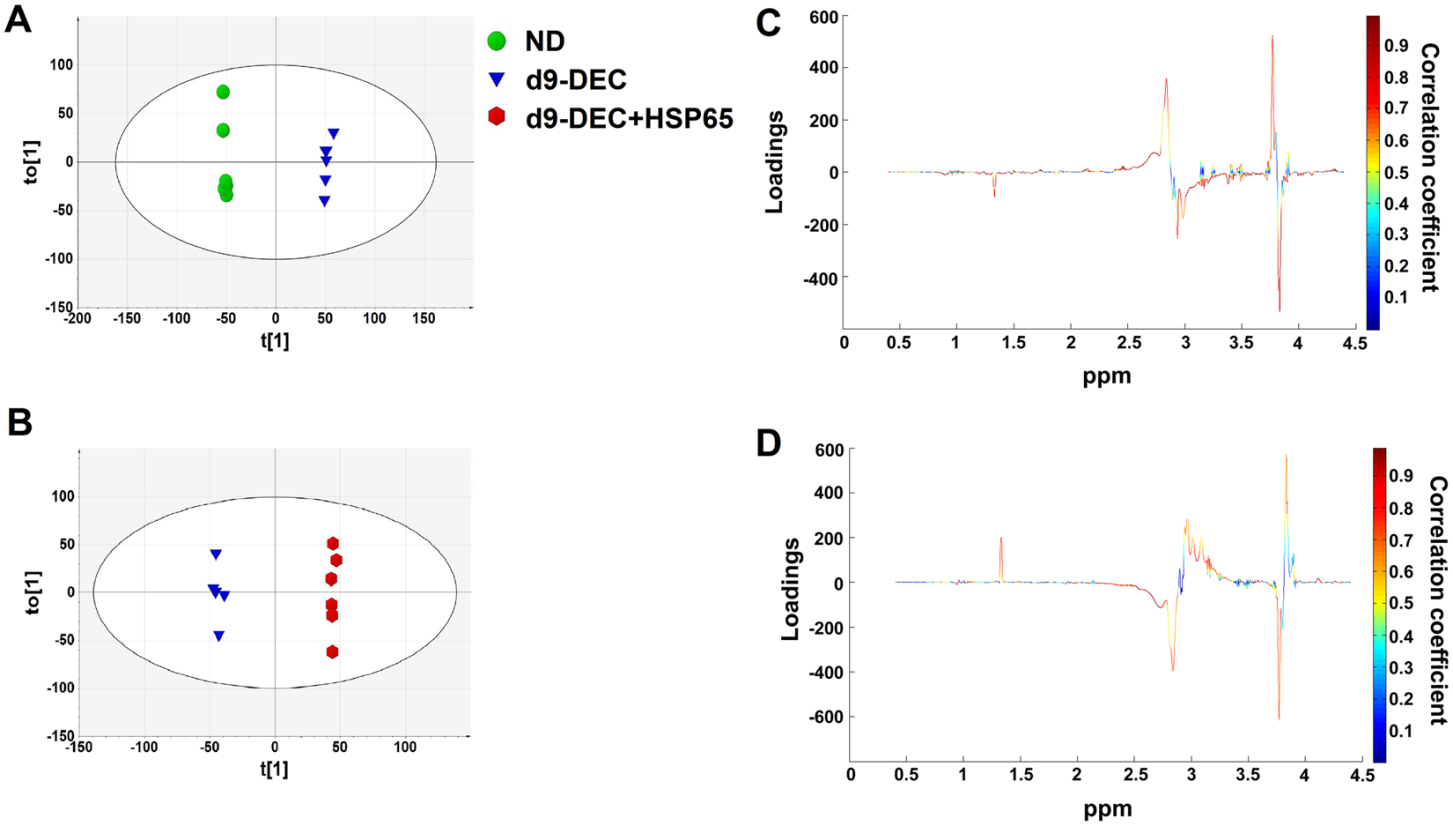
OPLS-DA analysis of culture media metabolic profiles of ND, d9-DEC and d9-DEC + HSP65. OPLS-DA of ND vs DEC [R2X = 73.1%, R2Y = 0.998, Q2 = 0.981 and CV-ANOVA score p = 0.00267644] (**A**) and DEC vs DEC + HSP65 [R2X = 73.6%, R2Y = 0.998, Q2 = 0.958 and CV-ANOVA score p = 4.33 × 10^−5^] (**B**). Coefficient color coded loading plots corresponding to OPLS-DA model of ND vs DEC (**C**) and DEC vs DEC + HSP65 (**D**) are represented.

In order to characterize the secreted metabolic flux of hESC culture, the media was analyzed every three days using 1H NMR followed by multivariate analysis. First, PCA was applied to visualize the inherent variance in the data. PCA could differentiate between day(d)0 and d3, d6 and d9 culture media metabolomic profiles of DEC and DEC + HSP65; however, we found overlaps between d3, 6 and 9 of DEC and DEC + HSP65 (Supplementary Figure S3A). Subsequently, PLS-DA showed a similar trend in class separation (Supplementary Figure S3B). Further, independent analysis of d0, 3, 6 and 9 of DEC and DEC + HSP65 was performed. While this analysis showed separation of all DEC groups from each other (Fig. 3A), d6 and 9 of DEC + HSP65 showed considerable overlaps (Fig. 3B). Considering these findings, HSP65 influences the metabolic changes associated with decidualization of the hESC. Next, metabolic profiles of only ND, d9-DEC and d9-DEC + HSP65 were included and analyzed. This analysis revealed good class discrimination between the groups indicating that metabolome of DEC + HSP65 differs from DEC (Fig. 3C). OPLS-DA models showed discrimination between ND, DEC and DEC + HSP65 (Fig. 4A and B).

For identification of significant metabolites contributing to class separation, S-plot [r ≥ ±0.60; Fig. 4C (ND vs DEC) and Fig. 4D (DEC vs DEC + HSP65)] and VIP plot (score ≥1) based on OPLS-DA models were generated. Similar to the endogenous metabolites identification, we used 2D NMR experiments (COSY and TOCSY) and literature for metabolites identification^18, 19^. The representative NMR spectra of media used in decidualized cells are shown in Supplementary Figure S2B and the identified metabolites indicated in Supplementary Table S2. Significantly higher levels of lactate, succinate, citrate, glutamine, alanine, isoleucine, phenylalanine, proline and threonine and lower levels of D-glucose, choline, 3-hydroxybutyrate and histidine in DEC compared with ND was observed (Table 1). DEC + HSP65 showed increased expression of acetate and alanine and decreased expression of D-glucose, lactate, citrate, myoinositol, choline, succinate, isoleucine, phenylalanine, proline and taurine as compared with DEC (Table 1).

### Correlation between endogenous metabolites and decidual secretome

**Figure 5:**
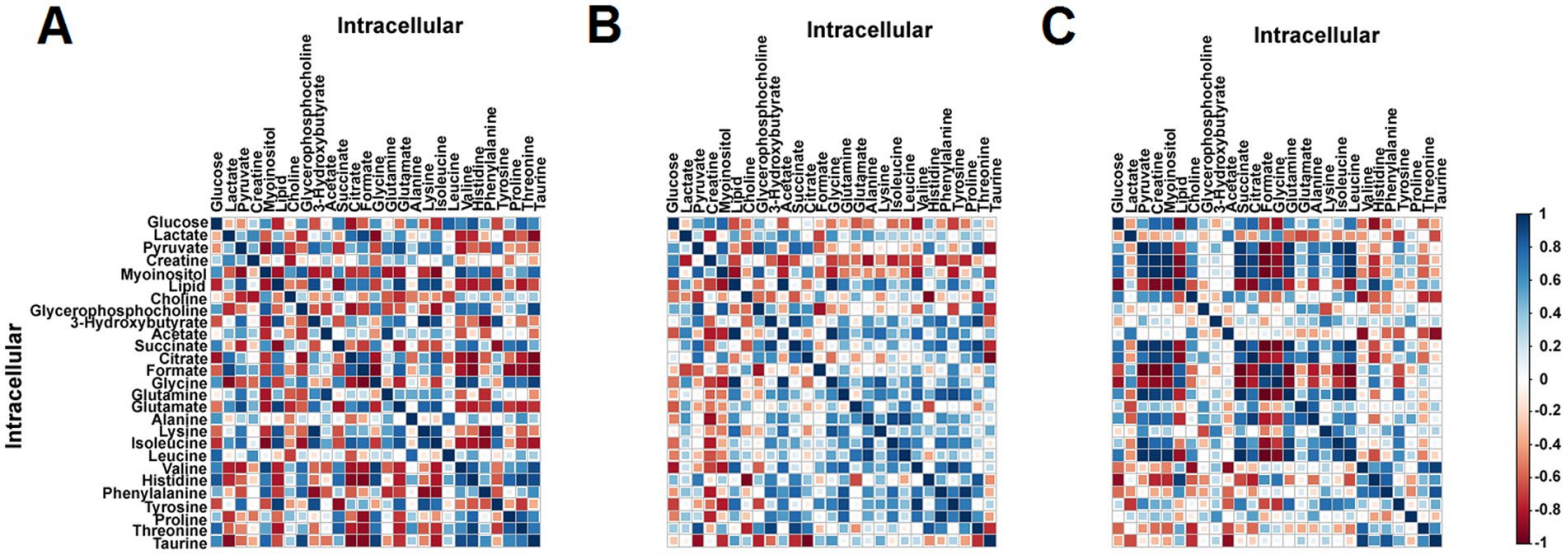
Differential correlation heatmap of intracellular metabolites using Pearson’s method. Metabolite-metabolite correlation of hESC (**A–C**) in ND, DEC and DEC + HSP65 is shown. Blue squares indicate significant positive correlations, white squares show non-significant correlations and red squares indicate significant negative correlations. Intermediate correlation coefficient values are indicated by size of the squares and varying shades of blue and red color.

Metabolite correlation heat maps were generated from intracellular metabolites data of ND, DEC and DEC + HSP65 (Fig. 5A–C). The observed pair-wise correlation of 25 metabolites present in the three groups showed a different trend in correlation pattern (both positive and negative) and is shown in Figure 5.

Glycolysis related metabolites showed a significant correlation with amino acids, choline, sugars, end-products of lipids and fatty acid oxidation in DEC + HSP65. Correlation between metabolites related to amino acid metabolism, tricarboxylic acid (TCA) cycle, end-products of lipids and fatty acid oxidation, and membrane metabolism suggests that HSP65 treatment during decidualization alters metabolites expression in a dependent manner. Similarly, correlation heat maps generated from spent media metabolites data of ND, DEC and DEC + HSP65 are shown in Supplementary Figure S4A–C. Here, we also explored the possible association of endogenous metabolites with decidual secretome. Endogenous metabolites significantly correlated with the decidual secretome (Supplementary Figure S5A–C), thereby suggesting that expression of metabolites in culture media is dependent on endometrial cells metabolism during decidualization with and without HSP65 treatment.

### HSP65 mediated metabolic pathways and networks

**Figure 6:**
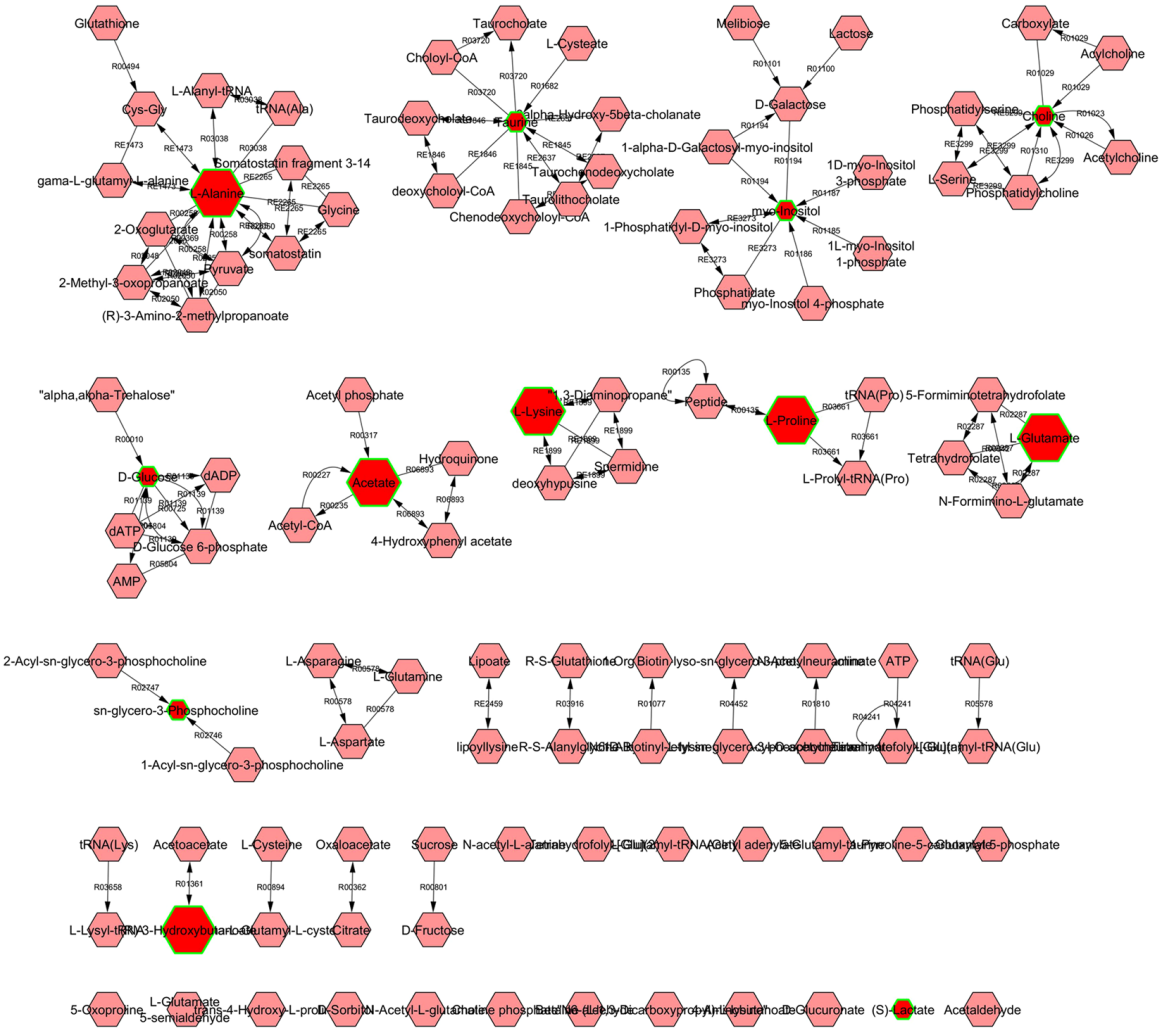
Markov clustering based reconstruction of HSP65 centered metabolic networks is represented. The clustering generates modules for each dysregulated intracellular metabolites in HSP65 treated decidualized cells. The input compound (red hexagon with green rim) and its related compounds (pink hexagon) and chemical reactions are indicated.

Metabolomic pathways analysis of significantly dysregulated intracellular and extracellular metabolites showed 26 (Supplementary Table S3) and 28 (Supplementary Table S4) altered pathways, respectively in HSP65 treated hESC during decidualization. Based on the impact score, taurine and hypotaurine metabolism, pyruvate metabolism and D-glutamine and D-glutamate pathways with changes in energy metabolism were observed to be majorly involved in decidualizaiton of hESC during HSP65 treatment (Supplementary Figures S6A and S7A). Next, HSP65-centered metabolic networks were constructed and visualized using Cytoscape based Metscape plug-in (Supplementary Figures S8A and S9A and Supplementary Tables S5 and S6). The resulting HSP65 mediated metabolic network consisted of 6 connected components with 112 nodes and 7 connected components with 108 nodes in cells and media, respectively, which indicated that majority of metabolites were interconnected (Supplementary Figures S6B and S7B). HSP65 centered network modules followed the power law distribution, which represents the specificity of generated network modules (Supplementary Figures S6C and S7C). High degree was observed with metabolites including pyruvate, L-glutamate, 2-oxoglutarate, D-glucose, acetate, L-alanine, L-lysine, choline and myoinositiol (Supplementary Figures S8B and S9B). This indicated that HSP65 affected pyruvate metabolism, Kreb’s cycle and amino acid metabolism with corresponding changes in energy metabolism. The network revealed that significantly dysregulated metabolites in cells treated with HSP65 involved in 15 different canonical metabolic pathways (Table 2), suggesting the effect of HSP65 in hESC metabolism during *in vitro* decidualization. For reducing the complexity of metabolic pathways and networks, network models generated from dysregulated intracellular and extracellular metabolites in HSP65 treated decidualized cells were reconstructed using Markov clustering (Fig. 6 and Supplementary Figure S10).

**Table 2:** List of HSP65 mediated metabolic pathways (both intracellular and extracellular) with average degree values.

Pathways	Degree	
	Cells	Media
Histidine metabolism	12.5	—
Vitamin B9 (folate) metabolism	10.4	—
Fructose and mannose metabolism	7.5	7.5
TCA cycle	7	6.3
Galactose metabolism	6	6
Glycine, serine, alanine and threonine metabolism	5.56	5.33
Lipoate metabolism	5	—
Vitamin H (biotin) metabolism	5	—
Urea cycle and metabolism of arginine, proline, glutamate, aspartate and asparagine	4.875	5.17
Glycolysis and Gluconeogenesis	4.43	5.29
Lysine metabolism	4.25	—
Glycerophospholipid metabolism	4.08	4.9
Methionine and cysteine metabolism	4	4
Bile acid biosynthesis	2.89	2.89
Phosphatidylinositol phosphate metabolism	2.89	2.89
Butanoate metabolism	1	6
Biopterin metabolism	—	9.75
Phytanic acid peroxisomal oxidation	—	5
Tyrosine metabolism	—	5.56
Valine, leucine and isoleucine degradation	—	2.8

## Discussion

Decidualization of stromal cells supports embryo implantation into the endometrium^7, 8, 20^. This process of increased cellular proliferation involves substantial metabolic processes and biosynthetic activity to achieve high energy demand during replication of all macromolecular components^21, 22^. Despite increasing evidence of enhanced glucose metabolism in endometrial decidualization^23–25^, the key regulatory pathways involving the metabolic homeostasis of hESC decidualization process is yet to be unraveled. In this study, for the first time, we explore the metabolic events underlying the transition from fibroblast stromal cells into decidual cells of the endometrium for an in-depth understanding of the implantation process at a molecular level.

Based on our compelling data indicative of aberrant molecular and morphological phenotype of the endometrium in dormant GTB women^4^, it is possible that the fundamental deficiency resides in the endometrial stromal cells. We, therefore, in the present study, focus our attention on endometrial stromal cells to further investigate the metabolic alterations responsible for reduced receptivity in women with dormant GTB. As we described earlier, Mycobacterial HSP65 hinders key molecular events associated with endometrial stromal cells decidualization necessary for endometrial receptivity^4^. We have also reported that compromised LIF mediated STAT3 signaling in hESC after HSP65 treatment is leading to poor decidualization^4^. In this perspective, we explored the metabolic reprogramming in hESC treated with Mycobacterial HSP65 during *in vitro* decidualization. Our findings indicate that HSP65 alters the metabolic networks of hESC decidualization, which are primarily related to TCA cycle, fatty acid biosynthesis, amino acid metabolism and energy metabolism (Supplementary Figures S6, S7, S8 and S9 and Table 2). In line with these findings, our earlier NMR-based metabolomic studies on dormant GTB women showed alterations in the intermediates of metabolic pathways related to energy metabolism and protein biosynthesis^17, 26^. Metabolic flux of the decidual cells of the endometrium showed decreased glucose expression with concurrent increase in lactate levels. This constitutive glucose uptake and lactate production regardless of oxygen availability in the decidual cells could be attributed to the Warburg effect or aerobic glycolysis^21^. A similar trend was observed in decidual secretome of hESC as compared to non-decidualized cells. It is well agreed upon that glycolytic intermediates majorly support the biochemical and energy demands of proliferating cells^22^. It is, therefore, logical to assume that most of the glucose utilized by decidualized endometrial cells is converted to lactate with part of it being utilized for oxidative phosphorylation. These findings are supported by studies reporting that decidual cells of the endometrium rely on high rate of glycolytic flux for the generation of ATP^24, 25^.

On treatment of hESC with HSP65 during *in vitro* decidualization, the metabolic reprogramming characterized by negligible lactate production and very low glucose concentration indicates a possible metabolic shift of most of the pyruvate to the TCA cycle via acetyl-CoA. This nutrient deprivation may be associated with a slow cell growth rate of hESC^27^.

The increased levels of TCA cycle intermediates, citrate and succinate in spent media of decidualized cells suggest their involvement in higher energy consumption for macromolecule synthesis. However, we could not observe any differences in the expression of these endogenous metabolites. Several reports suggest that highly proliferating cells generate energy through oxidative phosphorylation where nicotinamide adenine dinucleotide (NADH) donates electrons to electron transport chain in the mitochondria^27, 28^. While treating hESC with HSP65, we found decreased levels of citrate and succinate in the decidualsecretome. These observations further support increased TCA cycle activity and metabolic switch from glycolysis to oxidative phosphorylation in decidual endometrial cells upon HSP65 stimulation.

Conceivably, the increasing energy demand for decidual cells is also achieved by the breakdown of fatty acids (β-oxidation) and lipid metabolism^25^. Increased levels of 3-hydroxybutyrate and acetate in decidual cells under HSP65 stimuli probably contribute to mitochondrial ATP generation by providing substrates to TCA cycle. It is likely that the metabolic switch towards fatty acid oxidation (3-hydroxybutyrate, acetone and acetate) also occurs during transition from hESC to decidual cells for cell energy supply. While creatine supplies energy to the cells^29, 30^, increased utilization of creatine in decidual cells represents the high energy demand during decidualization process.

In addition to carbon source derived from glycolysis, lipid metabolites (choline, glycerophosphocholine and glycerol) provide substrates to produce ATP in decidual cells for maximal cell growth. Upon HSP65 stimulus, we observed decrease in choline and glycerophosphocholine suggesting a key role of choline metabolism in the process. Our findings are strengthened by previous reports where groups have shown that choline kinase enzyme phosphorylates intracellular choline to phosphocholine in various types of cancer including endometrial cancer^31–33^. It is also reported that decreased glycerophosphocholine/phosphocholine ratio occurs due to elevated phosphocholine and reduced glycerophosphocholine in cancer cells^34^. Owing to technical constraints, the expression of phosphocholine could not be ascertained. Down-regulation of myoinositol in HSP65 treated cells is also indicative of altered lipid metabolism. Various reports are in good agreement with our findings that decreased myoinositol level is associated with poor implantation and low pregnancy outcome^17, 35, 36^.

Amino acid metabolism is a highly regulated process and associated with protein biosynthesis and gluconeogenesis^14, 37^. Cells get access to various catabolic pathways for degradation of macromolecules when nutrients are deprived. Cellular uptake of amino acids from extracellular surface occurs through surface transporters activated by stimuli/signaling system^21, 38^. The branched chain amino acids (isoleucine, valine and leucine) in decidual cells provide anaplerotic flux for replenishing TCA cycle intermediates and maintaining homeostasis of cellular metabolism^38–40^. It is also reported that cellular degradation of leucine is involved in supply of acetyl-CoA^41^. A significant increase in alanine, lysine, histidine, tyrosine, phenylalanine and proline during decidualization process implies that these amino acids are getting oxidized for the energy supply instead of undergoing protein biosynthesis. Further, concomitant decrease in glucose and increase in alanine show the possible involvement of glucose-alanine cycle in decidualization for energy supply under anaerobic cellular respiration^42^.

Despite glucose being the major source of acetyl-CoA for fatty acid synthesis in cultured cells^38, 43^, glutamine and acetate have been established as alternative carbon source during hypoxia^44–46^. During series of deamination and transamination reactions, glutamine gets converted into intracellular glutamate and α-ketoglutarate to support TCA cycle, which is also involved in amino acid biosynthesis^47^. These observations support our findings where increased intracellular glutamate in HSP65 treated cells was observed. Increased glutamine levels in spent media and decreased intracellular glutamate during endometrial decidualization represent the enhanced cellular uptake of glutamate and low conversion rate of glutamine. We also found taurine levels to be significantly less in HSP65 treated decidual cells. Low level of taurine in the endometrium is suggestive of poor implantation^48, 49^.

Summarizing, the present study demonstrates that Mycobacterial HSP65 alters the metabolic homeostasis of hESC decidualization, an essential step for successful implantation. This altered metabolic activity is majorly associated with energy metabolism with concurrent changes in lipid and amino acid metabolism. These findings provide insight into the interface between cellular metabolism and non-receptive status of the endometrium. Since HSP65 mediates aberrant decidualization of hESC via metabolic reprogramming, HSP65 and its metabolic targets may be of clinical relevance in diagnosing and/or treating GTB women with recurrent implantation failure. Importantly, our findings also offer knowledge of cellular metabolism in the endometrial physiology for improved understanding of biochemical changes during hESC decidualization.

## Methods

### Subject selection

Approval was obtained from the Institutional research ethics committee of the Institute of Reproductive Medicine, Kolkata and Indian Institute of Technology, Kharagpur, India prior to commencement of the study and all methods and experiments were performed in compliance with relevant guidelines and regulations. Endometrial biopsy samples were collected by dilation and curettage (D&C) from five regularly cycling, non-smoking healthy fertile women during proliferative phase (day 6–10) of regular menstrual cycle. All these women agreed to participate in the study and provided written informed consent. Women ages 20–40 years, body mass index ranging 19–25 and normal serum hormone profile were included in the study. All these women had no history of any chronic disease, endocrine disorder and were not under any medication/hormonal therapy since the past three months. In order to confirm that the participants were free of GTB, endometrial tissue samples were subjected to BACTEC-460 culture, hematoxylin and eosin (H&E) staining, Ziehl–Neelsen (ZN) staining and single nested PCR. All women tested negative for GTB. Further, women with sexually transmitted infections including *Chlamydia trachomatis*, *Neisseria gonorrhoeae*, *Trichomonas vaginalis*, *Treponema pallidum*, *Mycoplasma genitalium*, *Mycoplasma hominis*, *Ureaplasma urealyticum*, *Gardnerella vaginalis* and *Candida albicans*, bacterial vaginosis and viral infections (human immunodeficiency virus, herpes simplex virus type 2, human papillomavirus and hepatitis-B) and any associated pelvic pathology were excluded.

### Isolation of primary endometrial stromal cells and *in vitro* decidualization

Human endometrial stromal cells were isolated by enzymatic digestion, as described elsewhere^50–52^. Briefly, endometrial tissues were minced into small pieces and incubated in phenol red-free DMEM/F12 containing 0.25% type I collagenase (Sigma-Aldrich, St. Louis, Missouri, USA) for 60 min at 37 °C. The cell suspension was separated by successive filtrations through 100 μm and 40 μm cell strainer. Cells were layered over a Histopaque reagent to remove blood cells (Sigma-Aldrich, St. Louis, Missouri, USA). The resultant single cells were cultured in DMEM/F12 medium (Invitrogen, San Diego, California, USA) supplemented with 10% charcoal-stripped fetal bovine serum (FBS), 1× insulin-transferrin-selenium (Invitrogen,San Diego, California, USA) and 1% antibiotic-antimycotic (penicillin, streptomycin and amphotericin-B).

To induce decidualization, cells (at 70–80% confluency) were treated with 1 μM MPA and 50 μM cAMP (Sigma-Aldrich, St. Louis, Missouri, USA) every 48–72 h for 7–9 days. *In vitro* decidualization was performed as described previously^4, 53^. Initially, hESC were treated with four different concentrations of HSP65 (0.01, 0.1, 1 and 5 µg/ml). While treatment of hESC with 1 and 5 µg/ml of HSP65 during *in vitro* decidualization showed complete detachment of cells from plates after 5 days of treatment, there was no change observed with treatment of 0.01 µg/ml HSP65. Hence, 0.1 µg/ml of HSP65 was used for the present study. Cells were exposed to 0.1 μg/ml of HSP65 or vehicle (PBS) throughout the decidualization process. Morphological changes of the isolated endometrial stromal cells were monitored using a phase contrast microscope (Olympus Optical Co., Ltd, Tokyo, Japan). All experiments were carried out with early passage hESC (≤passage 5).

### Quantitative reverse transcriptase PCR

Total RNA was isolated from hESC and reverse transcribed using cDNA synthesis kit as per manufacturer’s instructions (Bio-Rad, Hercules, California, USA). Quantitative Real time PCR was performed using SYBR Green Supermix (Bio-Rad, Hercules, California, USA), as described elsewhere^4^. The average of housekeeping genes (GAPDH, L19, LDHA and RPS18) was used to normalize the transcript abundance of prolactin and IGFBP1. Sequences of these primer sets are provided in Supplementary Table S1.

### Immunofluorescence Study

Immunofluorescence was used to study the expression profiles of hESC before and after treatment with HSP65 protein. Briefly, cells were cultured on lysine coated cover slips in 12 well cell culture plates till confluence was reached. After treatment with HSP65 for a specific time period, the cells were fixed with 4% formaldehyde solution for 30 min. Following blocking with 1% bovine serum albumin and permeablization with 0.1% Triton X-100, cells were incubated with primary antibodies (sc-20726 (prolactin) and sc-13097 (IGFBP1); Santa Cruz Biotechnology, Santa Cruz, California, USA) at 4 °C overnight. Alexa Fluor 488 Goat anti-rabbit secondary antibody (Invitrogen, San Diego, California, USA) was added and visualized under fluorescence microscopy.

### Extraction of metabolites from cells and media

Metabolites were extracted from cells, as previously described^54, 55^. Endometrial stromal cells were cultured and decidualized with and without HSP65 treatment (6 replicates) in 6 well plates for 9 days. Following decidualization, media was replaced with FBS-free DMEM medium and incubated for 24 h prior to harvest. Five volumes of 60% methanol with 0.85% (w/v) ammonium bicarbonate were added to the plates for quenching cellular metabolism at a minimum temperature of −40 °C. The quenched cells were transferred to tubes and centrifuged. The pellet was rinsed with prechilled 80% methanol and centrifuged for 5 min at 2,000 × g. The supernatant was retained in every step and the extraction process repeated. The pooled extracted metabolites were dried using Speed Vac concentrator (Eppendorf concentrator plus, Eppendorf UK, Ltd., Cambridge, U.K.) and stored at −80 °C until NMR analysis.

Extraction of metabolites from spent media was performed using methanol:chloroform extraction^55^. Media from hESC culture was collected during d0, 3, 6 and 9. Media was mixed with 3 volumes of prechilled methanol:chloroform solution (2:1) and vortexed for 30 sec. Following the addition of 600 μL prechilled methanol, the mixer was centrifuged at 13,000 rpm for 20 min. The collected aqueous layer was dried using Speed Vac concentratorand stored at −80 °C.

### NMR experiment and data processing

1H NMR﻿ spectra of all the extracted metabolites were acquired at 298 K on a 700 MHz Bruker Avance AV III spectrometer using NOESYGPPR 1D pulse sequence for efficient water signal suppression. The metabolite extracts were resuspended in 600 µl of D2O with 3-(trimethylsilyl) propionic-2, 2, 3, 3, d4 acid (TSP) and loaded into 5 mm NMR tubes. 1H NMR spectra with WATERGATE solvent suppression were recorded with 256 transients, spectral width of 14,367.8 Hz, 16384 data points, 1.5 sec relaxation delay and an acquisition time of 1.14 sec. 2D NMR spectra were obtained after the 1D NMR spectra using TOCSY and COSY for metabolite identification purposes.

Following manual phase and baseline correction, the 1D spectra were referenced to TSP (δ = 0.0 ppm) in MestReNova version 7.1.0 (Mestrelab Research, Santiago de Compostela, Spain). NMR spectra were aligned by R-based recursive segment-wise peak alignment using the R/Bioconductor package mQTL.NMR for minimizing the chemical shift variations. After removal of water signal region (δ 4.5–5.10 ppm), the spectral region of *δ* 0.5–4.5 and *δ* 5.10–9.0 were subjected to further multivariate and univariate analysis. Spectra were normalized using constant sum normalization and scaled with unit variance scaling (SIMCA 13.0.2, Umetrics, Sweden).

### Chemometrics and data analysis

Multivariate analysis was performed to the datasets as described elsewhere^17, 26^. PCA, PLS-DA and OPLS-DA were generated using SIMCA 13.0.2 (Umetrics, Sweden). Robustness and validation of the OPLS-DA model was validated by R2, Q2 and CV-ANOVA score. S-line plot and VIP score were used to identify significant regions discriminating the groups. Spectral integration was applied to the identified significant variables (MestReNova version 7.1.0, Mestrelab Research, Santiago de Compostela, Spain). The integral values of identified metabolites were normalized with constant sum normalization and subjected to univariate analysis. Media constitutes were subtracted in case of NMR analysis of culture media. Correlation heatmaps were generated to identify the relationship between the metabolites using R statistical packages version 3.2.2 (R Foundation for Statistical Computing, Vienna, Austria; http://www.R-project.org/).

### Metabolic pathway and Network analysis

Pathway enrichment and topology analysis of metabolites integral values were performed using metabolomics pathway analysis^56^ (Metaboanalyst 3.0; MetPA, http://metpa.metabolomics.ca). The algorithms selected for pathway enrichment analysis and topological analysis are global test and relative betweenness centrality, respectively. Metabolic interactions networks were constructed using Metscape2, a Cytoscape based plug-in^﻿57^ (http://www.metscape.ncibi.org/tryplugin.html). The complex biological networks were analyzed and reconstructed using Markov clustering based module analysis^58^.

### Statistical analysis

Statistical significance of data between the groups was attained using Student’s t-test, Mann–Whitney U test and one-way analysis of variance, as applicable (GraphPad Prism version 5.00 for Windows, GraphPad Software, San Diego, California, USA). Statistical significance was set to p < 0.05.

## Electronic supplementary material

Supplementary information
