# Supplementary material for: Mycobacterial heat shock protein 65 mediated metabolic shift in decidualization of human endometrial stromal cells
Source: Sci Rep. 2017 Jun 21;7:3942. doi: 10.1038/s41598-017-04024-w (PMC5479817; doi:10.1038/s41598-017-04024-w)
Supplement: Supplementary file 1 — Supplementary information [file 41598_2017_4024_MOESM1_ESM.pdf]

SUPPLEMENTARY INFORMATION

Mycobacterial heat shock protein 65 mediated metabolic shift in decidualization of human endometrial stromal cells

Elavarasan Subramani[1], Arun Prabhu Rameshbabu[1], Manivannan Jothiramajayam[2], Bhuvaneshwaran Subramanian[1], Debangana Chakravorty[3], Gunja Bose[4], Mamata Joshi[5], Chaitali Datta Ray[6], Indrani Lodh[4], Ratna Chattopadhyay[4], Sudipto Saha[3], Anita Mukherjee[2], Santanu Dhara[1], Baidyanath Chakravarty[4], Koel Chaudhury[1]*

SUPPLEMENTARY MATERIAL

Table S1. Primer sequences of genes used for this study

Gene		Sequence (5'-3')	Amplicon length (bp)
Prolactin	Forward	ATCATCTGGTCACGGAAGTACG	83
	Reverse	GGTTTGCTCCTCAATCTCTACAG	
IGFPB1	Forward	TTTTACCTGCCAAACTGCAACA	108
	Reverse	CCCATTCCAAGGGTAGACGC	
GAPDH	Forward	CTGGGCTACACTGAGCACC	101
	Reverse	AAGTGGTCGTTGAGGGCAATG	
RPS18	Forward	ATCACCATTATGCAGAATCCACG	93
	Reverse	GACCTGGCTGTATTTTCCATCC	
L19	Forward	GGGCATAGGTAAGCGGAAGG	149
	Reverse	TCAGGTACAGGCTGTGATACA	
LDHA	Forward	ATGGCAACTCTAAAGGATCAGC	86
	Reverse	CCAACCCCAACAACTGTAATCT	

Table S2. List of identified metabolites in endometrial stromal cells and culture media with their spin multiplicities

Metabolites	Structure	Moieties	δ ^{1}H (ppm) and multiplicity	KEGG ID	Samples
1-Methylhistidine	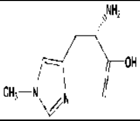	αCH, βCH	7.67 (s), 7.00 (s)	C01152	M
3-hydroxybutyric acid	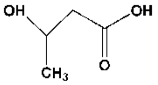	γCH_3, ½ αCH_2, ½ αCH_2	1.204 (d), 2.314 (m), 2.414 (m),	C01089	C, M
Acetic acid	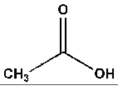	CH_3	1.91 (s)	C00033	C, M
Alanine	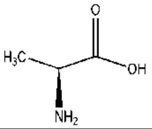	βCH_3, αCH	1.46 (d), 3.76 (q)	C00041	C, M
Choline	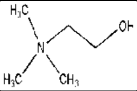	$N(CH_3)_3$, NCH_2, OCH_2	3.189 (s), 3.507 (dd), 4.056 (ddd)	C00114	C, M
Citric acid	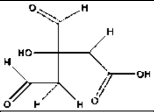	½ CH_2, ½ CH_2	2.65 (d), 2.53 (d)	C00158	C, M
Creatine	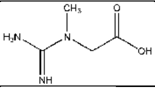	CH_2, CH_3	3.92 (s), 3.02 (s)	C00300	C
Formic acid	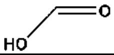	CH	8.44 (s)	C00058	C
Glucose and mixed aminoacids		(αCH)-resonances	3.3-3.9	-	C, M
Glutamic acid	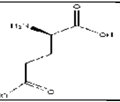	γCH_2, αCH	2.36 (m), 3.77 (m)	C00217	C, M
Glutamine	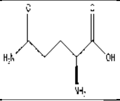	βCH_2, γCH_2, αCH	2.125 (m), 2.446 (m), 3.766 (t)	C00064	C, M
Glycerophosphocholine	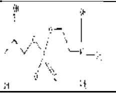	-CH	3.20 (s)	C00670	C
Glycine	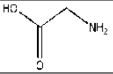	CH_2	3.54 (s)	C00037	C, M
Histidine	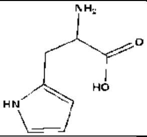	αCH, 5CH, 3CH	3.98 (dd), 7.09 (d), 7.90 (d)	C00135	C, M
Isoleucine	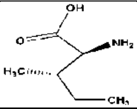	δCH_3, γCH_2, γ'CH_2	0.926(t), 1.248(m), 1.457(m),	C00407	C, M

Lactic acid	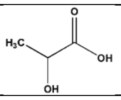	βCH$_3$, αCH	1.32 (d), 4.10 (q)	C00186	C, M
Leucine	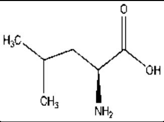	δCH$_3$, βCH$_2$, αCH	0.948 (t), 1.700 (m), 3.722 (m)	C00123	C, M
Lipid		CH$_3$, (CH$_2$)$_n$, -CH=CH-	0.88 (m broad), 1.30 (m broad), 5.3 (m broad)	-	C
Lysine	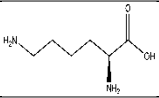	γCH$_2$, αCH	1.71 (m), 3.74 (t)	C00047	C, M
Myoinositol	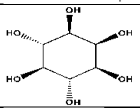	H1/H3 CH, H4/H6 CH	3.524 (dd), 3.613 (t),	C00137	C, M
Oxypurinol	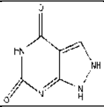	δCH$_3$, βCH$_2$	8.32	C07599	M
Phenylalanine	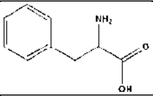	αCH,2, 4CH, 5CH	3.98 (dd), 7.32 (d), 7.42 (m)	C00079	C, M
Proline	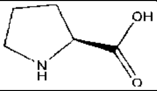	β'CH$_2$, δCH$_2$, δ'CH$_2$, αCH	2.34 (m), 3.33 (dt), 3.41 (dt), 4.12 (dd)	C00148	C, M
Pyruvate	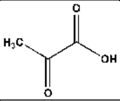	CH$_3$	2.46 (s)	C00022	C, M
Succinic acid	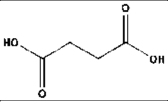	2*CH$_2$	2.393 (s)	C00042	C, M
Taurine	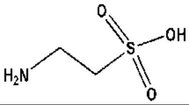	CH$_2$SO$_3$, NCH$_2$	3.25(t), 3.42(t),	C00245	C, M
Threonine	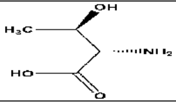	γCH$_3$, αCH, βCH	1.316 (d), 3.575 (d), 4.244(m)	C00188	C, M
Tyrosine	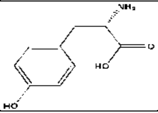	αCH, 6CH	6.877 (m), 7.170 (m)	C00082	C, M
Valine	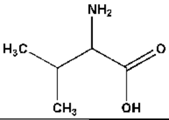	γCH$_3$, γCH$_3$,	0.976 (d), 1.029 (d)	C00183	C, M
α-glucose	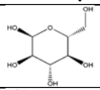	1CH	5.22 (d)	C00267	C, M
β-Glucose	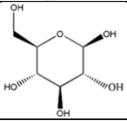	1CH	4.65 (d)	C00221	C, M

s-singlet; d-doublet; t-triplet; q-quartet; m-multiplet; dd-doublet of doublet; dt-doublet of triplet; C-Intracellular metabolites; M-Extracellular metabolites

Table S3: Metabolic pathway analysis shows the HSP65 mediated pathways in endometrial stromal cells during decidualization

Pathway Name	Total	Hits	p	-log(p)	Holm p	FDR	Impact
Taurine and hypotaurine metabolism	20	3	1.30E-06	13.55	3.39E-05	1.56E-05	0.36331
D-Glutamine and D-glutamate metabolism	11	1	0.026215	3.6414	0.067496	0.026215	0.3262
Pyruvate metabolism	32	2	3.24E-05	10.339	6.80E-04	1.40E-04	0.23703
Lysine degradation	47	1	9.91E-04	6.9171	0.016098	0.00184	0.14675
Arginine and proline metabolism	77	1	0.017973	4.0189	0.067496	0.019471	0.10231
Lysine biosynthesis	32	1	9.91E-04	6.9171	0.016098	0.00184	0.09993
Alanine, aspartate and glutamate metabolism	24	1	1.83E-06	13.212	4.57E-05	1.56E-05	0.05698
Aminoacyl-tRNA biosynthesis	75	3	2.40E-06	12.94	5.52E-05	1.56E-05	0.05634
Sulfur metabolism	18	1	1.87E-04	8.5829	0.003558	6.09E-04	0.03307
Glycerophospholipid metabolism	39	2	0.004266	5.4572	0.051185	0.007074	0.03105
Starch and sucrose metabolism	50	1	0.005445	5.213	0.054453	0.007079	0.01703
Primary bile acid biosynthesis	47	1	9.47E-04	6.9623	0.016098	0.00184	0.00822
Vitamin B6 metabolism	32	1	0.026215	3.6414	0.067496	0.026215	0.00798
Butanoate metabolism	40	1	0.011249	4.4874	0.067496	0.013295	0.0048
Selenoamino acid metabolism	22	2	9.68E-06	11.546	2.13E-04	5.03E-05	0.00321
Galactose metabolism	41	1	0.005445	5.213	0.054453	0.007079	0.00276
Glycolysis or Gluconeogenesis	31	3	1.09E-04	9.1243	0.00218	4.05E-04	4.60E-04
Cysteine and methionine metabolism	56	1	1.83E-06	13.212	4.57E-05	1.56E-05	0
Propanoate metabolism	35	1	7.71E-04	7.168	0.013876	0.00184	0
Nitrogen metabolism	39	1	9.47E-04	6.9623	0.016098	0.00184	0
Biotin metabolism	11	1	9.91E-04	6.9171	0.016098	0.00184	0
Ether lipid metabolism	23	1	0.004354	5.4368	0.051185	0.007074	0
Pentose phosphate pathway	32	1	0.005445	5.213	0.054453	0.007079	0
Amino sugar and nucleotide sugar metabolism	88	1	0.005445	5.213	0.054453	0.007079	0
Synthesis and degradation of ketone bodies	6	1	0.011249	4.4874	0.067496	0.013295	0
Glycine, serine and threonine metabolism	48	1	0.015882	4.1425	0.067496	0.017954	0

Supplementary Table S4: Metabolic pathway analysis of significantly dysregulated metabolites in spent culture media of endometrial stromal cells during decidualization shows the effect of HSP65 on metabolic pathways

Pathway Name	Total	Hits	p	-log(p)	Holm p	FDR	Impact
Taurine and hypotaurine metabolism	20	3	0.003033	5.7982	0.03943	0.004735	0.36331
Pyruvate metabolism	32	2	0.008081	4.8182	0.042786	0.008703	0.23703
Inositol phosphate metabolism	39	1	5.50E-06	12.11	1.54E-04	7.70E-05	0.13703
Phenylalanine metabolism	45	2	1.04E-04	9.1728	0.002375	3.63E-04	0.11906
Arginine and proline metabolism	77	1	9.57E-05	9.2546	0.002296	3.63E-04	0.10231
Citrate cycle (TCA cycle)	20	2	5.72E-04	7.4668	0.008577	0.001067	0.07773
Alanine, aspartate and glutamate metabolism	24	2	0.003804	5.5718	0.042786	0.004735	0.05698
Sulfur metabolism	18	1	0.007153	4.9402	0.042786	0.008345	0.03307
Glycerophospholipid metabolism	39	1	0.0038	5.5727	0.042786	0.004735	0.0212
Butanoate metabolism	40	1	1.03E-04	9.1783	0.002375	3.63E-04	0.01774
Starch and sucrose metabolism	50	1	2.53E-04	8.281	0.004686	5.46E-04	0.01703
Valine, leucine and isoleucine biosynthesis	27	1	5.84E-05	9.7487	0.001518	3.63E-04	0.01325
Primary bile acid biosynthesis	47	1	0.023382	3.7558	0.046569	0.023382	0.00822
Glyoxylate and dicarboxylate metabolism	50	2	5.72E-04	7.4668	0.008577	0.001067	0.00326
Selenoamino acid metabolism	22	2	0.003819	5.5679	0.042786	0.004735	0.00321
Galactose metabolism	41	2	2.47E-04	8.3077	0.004686	5.46E-04	0.00276
Propanoate metabolism	35	2	0.008039	4.8235	0.042786	0.008703	0.00134
Phenylalanine, tyrosine and tryptophan biosynthesis	27	1	0.00389	5.5494	0.042786	0.004735	6.20E-04
Glycolysis or Gluconeogenesis	31	3	2.32E-04	8.3698	0.004635	5.46E-04	4.60E-04
Ascorbate and aldarate metabolism	45	1	5.50E-06	12.11	1.54E-04	7.70E-05	0
Valine, leucine and isoleucine degradation	40	1	5.84E-05	9.7487	0.001518	3.63E-04	0
Tyrosine metabolism	76	1	1.03E-04	9.1783	0.002375	3.63E-04	0
Pentose phosphate pathway	32	1	2.53E-04	8.281	0.004686	5.46E-04	0
Amino sugar and nucleotide sugar metabolism	88	1	2.53E-04	8.281	0.004686	5.46E-04	0
Aminoacyl-tRNA biosynthesis	75	4	0.003566	5.6364	0.042786	0.004735	0
Glycine, serine and threonine metabolism	48	1	0.0038	5.5727	0.042786	0.004735	0
Cysteine and methionine metabolism	56	1	0.003837	5.5632	0.042786	0.004735	0
Nitrogen metabolism	39	2	0.023285	3.76	0.046569	0.023382	0

Table S5. Compounds and their network parameters related to the altered metabolites in endometrial stromal cells treated with HSP65 during decidualization

Average Shortest PathLength	Betweenness Centrality	canonical Name	Category	Closeness Centrality	Clustering Coefficient	Compound.approximate MW	Compound.casnum	Compound.Cell_CvsHSP.Fold change	Compound.Cell_CvsHSP. p value	Compound.cid	Compound formula	Compound mw	Compound pubchemcid	Degree	direction	Eccentricity	name	Neighborhood Connectivity	Number Of Directed Edges	Number Of Undirected Edges	Partner Of Multi Edged Node Pairs	Radiality	Stress	Topological Coefficient
2.67272727	0	ATP	Compound	0.37414966	1	507	56-65-5			C00002	C10H16N5O13	507.181023	5957	2		4	C00002	16	1	1	0	0.721212	0	0.53333333
4.32727273	0	Peptide	Compound	0.23109244	0	0	NULL			C00012	C2H4NO2R(C2	0		3		6	C00012	5	2	0	0	0.445455	0	0
3.64285714	0	AMP	Compound	0.2745098	1	347	61-19-8			C00020	C10H14N5O7P	347.221221	6083	2		6	C00020	7.5	1	1	0	0.559524	0	0.68181818
2.45454545	0.03636364	Pyruvate	Compound	0.40740741	0.66666667	88	127-17-3			C00022	C3H4O3	88.06206	1060	9		4	C00022	10.16666667	7	2	2	0.757576	138	0.27027027
3.8	0	Acetyl-CoA	Compound	0.26315789	0	809	72-89-9			C00024	C23H38N7O17	809.570803	6302	2		6	C00024	12	2	0	1	0.533333	0	0
1.70909091	0.78338945	L-Glutamate	Input Compound	0.58510638	0.05977011	147	56-86-0	1.21	0.0262	C00025	C5H9NO4	147.12926	33032	39	up	3	C00025	3.13333333	28	11	5	0.881818	2612	0.08529412
2.21818182	0.05499439	2-Oxoglutarate	Compound	0.45081967	0.61904762	146	328-50-7			C00026	C5H6O5	146.09814	51	11		4	C00026	9.85714286	8	3	1	0.79697	336	0.24642857
2.71428571	0.48280423	D-Glucose	Input Compound	0.36842105	0.12727273	180	50-99-7	0.804	0.0054	C00031	C6H12O6	180.15588	5793	14	down	5	C00031	2.45454545	10	4	2	0.714286	400	0.20661157
2.81818182	0.33131313	Acetate	Input Compound	0.35483871	0.06060606	60	64-19-7	1.47	2.00E-04	C00033	C2H4O2	60.05196	176	14	up	5	C00033	1.91666667	10	4	2	0.69697	1284	0.14393939
2.21818182	0.34747475	Oxaloacetate	Compound	0.45081967	0.4	132	328-42-7			C00036	C4H4O5	132.07156	970	5		4	C00036	11.2	3	2	0	0.79697	1392	0.26666667
3.27272727	0	Glycine	Compound	0.30555556	1	75	56-40-6			C00037	C2H5NO2	75.0666	750	3		5	C00037	6.33333333	1	2	0	0.621212	0	0.48717949
2.32727273	0.22581369	L-Alanine	Input Compound	0.4296875	0.20512821	89	56-41-7	1.66	1.00E-04	C00041	C3H7NO2	89.09318	5950	16	up	4	C00041	5.61538462	11	5	2	0.778788	920	0.14968815
1	0.86666667	L-Lysine	Input Compound	1	0.13333333	146	56-87-1	1.33	0.001	C00047	C6H14N2O2	146.18756	5962	11	up	1	C00047	2.2	7	4	1	1	78	0.23333333
2.4	0.00942761	L-Aspartate	Compound	0.41666667	0.6	133	56-84-8			C00049	C4H7NO4	133.10268	5960	6		4	C00049	9.6	4	2	1	0.766667	56	0.3
2.65454545	0	Glutathione	Compound	0.37671233	1	307	70-18-8			C00051	C10H17N3O6S	307.32348	124886	2		4	C00051	17	2	0	0	0.724242	0	0.5483871
3.89285714	0	Carboxylate	Compound	0.25688073	1	44	NULL			C00060	CO2R	0		2		6	C00060	5.5	1	1	0	0.517857	0	0.61111111
2.65454545	0	L-Glutamine	Compound	0.37671233	1	146	56-85-9			C00064	C5H10N2O3	146.1445	5961	5		4	C00064	12.66666667	4	1	1	0.724242	0	0.42222222
3.85714286	0	L-Serine	Compound	0.25925926	1	105	56-45-1			C00065	C3H7NO3	105.09258	5951	3		6	C00065	5.33333333	2	1	0	0.52381	0	0.59259259
3.8	0	Acetaldehyde	Compound	0.26315789	0	44	75-07-0			C00084	C2H4O	44.05256	177	2		6	C00084	12	2	0	1	0.533333	0	0
3.64285714	0	Sucrose	Compound	0.2745098	1	342	57-50-1			C00089	C12H22O11	342.29648	5988	2		6	C00089	6.5	2	0	0	0.559524	0	0.59090909
3.57142857	0.0026455	D-Glucose 6-phosphate	Compound	0.28	0.66666667	260	56-73-5			C00092	C6H13O9P	260.135781	208	6		6	C00092	4.75	4	2	1	0.571429	4	0.43181818
3.64285714	0	D-Fructose	Compound	0.2745098	1	180	57-48-7			C00095	C6H12O6	180.15588		2		6	C00095	6.5	1	1	0	0.559524	0	0.59090909
2.67272727	0	L-Cysteine	Compound	0.37414966	1	121	52-90-4			C00097	C3H7NO2S	121.15818	5862	2		4	C00097	16	1	1	0	0.721212	0	0.53333333
2.63636364	6.73E-04	Tetrahydrofolate	Compound	0.37931034	0.66666667	445	135-16-0			C00101	C19H23N7O6	445.42922	1129	5		4	C00101	9.5	3	2	1	0.727273	4	0.31666667
2.96428571	0.38095238	Choline	Input Compound	0.3373494	0.13888889	104	62-49-7	0.579	0.0159	C00114	C5H14NO	104.17076	305	12	down	5	C00114	2.33333333	9	3	3	0.672619	328	0.25
1.8	0	Biotin	Compound	0.55555556	1	244	58-85-5			C00120	C10H16N2O3S	244.31064	171548	2		2	C00120	6	1	1	0	0.6	0	0.6
2.39285714	0.49603175	D-Galactose	Compound	0.41791045	0.3	180	59-23-4			C00124	C6H12O6	180.15588	439357	6		4	C00124	5	3	3	1	0.767857	420	0.26315789
3.60714286	0	dATP	Compound	0.27722772	1	491	1927-31-7			C00131	C10H16N5O12	491.181623	15993	3		6	C00131	6	2	1	0	0.565476	0	0.54545455
2.25	0.64814815	myo-Inositol	Input Compound	0.44444444	0.07142857	180	87-89-8	0.516	0.0113	C00137	C6H12O6	180.15588	892	8	down	3	C00137	2.125	6	2	0	0.791667	558	0.18055556
3.34545455	0.14074074	L-Proline	Input Compound	0.29891304	0.1	115	147-85-3	1.43	0.018	C00148	C5H9NO2	115.13046	145742	6	up	5	C00148	1.6	5	1	1	0.609091	426	0.33333333
2.65454545	0	L-Asparagine	Compound	0.37671233	1	132	70-47-3			C00152	C4H8N2O3	132.11792	6267	3		4	C00152	12.66666667	2	1	0	0.724242	0	0.42222222
3.14285714	0.05291005	Phosphatidylcholine	Compound	0.31818182	0.66666667	2049	8002-43-5			C00157	C10H18NO8PR	0	452110	5		5	C00157	4.75	4	1	1	0.642857	80	0.43181818
3	0	Citrate	Compound	0.33333333	1	192	77-92-9			C00158	C6H8O7	192.12352	311	2		5	C00158	8.5	2	0	0	0.666667	0	0.56666667
1	0	Acetoacetate	Compound	1	0	102	541-50-4			C00164	C4H6O3	102.08864	96	1		1	C00164	1	1	0	0	1	0	0
3.43636364	0	(S)-Lactate	Input Compound	0.29100529	0	90	79-33-4	0.891	8.00E-04	C00186	C3H6O3	90.07794	107689	2	down	5	C00186	6	2	0	1	0.593939	0	0
3.21428571	0	D-Glucuronate	Compound	0.31111111	0	194	12/3/1956			C00191	C6H10O7	194.1394	444791	1		4	C00191	8	1	0	0	0.630952	0	0
3.60714286	0	dADP	Compound	0.27722772	1	411	NULL			C00206	C10H15N5O9P	411.201722	188966	3		6	C00206	6	2	1	0	0.565476	0	0.54545455
3.8	0	Acetyl phosphate	Compound	0.26315789	0	140	NULL			C00227	C2H5O5P	140.031861	186	1		6	C00227	12	1	0	0	0.533333	0	0
3.03571429	0	Lactose	Compound	0.32941176	1	342	63-42-3			C00243	C12H22O11	342.29648	84571	2		5	C00243	8	2	0	0	0.660714	0	0.61538462
1	0.91111111	Taurine	Input Compound	1	0.08888889	125	107-35-7	0.626	9.00E-04	C00245	C2H7NO3S	125.14688	1123	10	down	1	C00245	1.8	6	4	0	1	82	0.2
3.78181818	0	N-Acetylneuraminate	Compound	0.26442308	1	309	131-48-6			C00270	C11H19NO9	309.26986	439197	2		6	C00270	7	1	1	0	0.536364	0	0.58333333
1.7	0	Spermidine	Compound	0.58823529	1	145	124-20-9			C00315	C7H19N3	145.24586	1102	3		2	C00315	5.33333333	2	1	0	0.65	0	0.53333333
2.69090909	0	4-Aminobutanoate	Compound	0.37162162	0	103	56-12-2			C00334	C4H9NO2	103.11976	119	1		4	C00334	30	1	0	0	0.718182	0	0
2.47272727	0	2-Methyl-3-oxopropanoate	Compound	0.40441176	1	102	NULL			C00349	C4H6O3	102.08864	296	6		4	C00349	12.2	4	2	1	0.754545	0	0.32105263
2.53571429	0.45238095	Phosphatidate	Compound	0.3943662	0.33333333	596	NULL			C00416	C5H7O8PR2	0	5873088	4		4	C00416	5.75	2	2	0	0.744048	418	0.33823529
2.65454545	0	N-Formimino-L-glutamate	Compound	0.37671233	1	174	NULL			C00439	C6H10N2O4	174.1546	13160	3		4	C00439	12.33333333	2	1	0	0.724242	0	0.41111111
1.9	0	"N6-(L-1,3-Dicarboxypropyl)-L-lysine"	Compound	0.52631579	0	276	997-68-2			C00449	C11H20N2O6	276.2863	160556	2		2	C00449	10	2	0	1	0.55	0	0
1.9	0	L-Cysteate	Compound	0.52631579	0	169	13100-82-8			C00506	C3H7NO5S	169.15638	25701	1		2	C00506	10	1	0	0	0.55	0	0
3.78181818	0	Hydroquinone	Compound	0.26442308	1	110	123-31-9			C00530	C6H6O2	110.11064	785	2		6	C00530	7	1	1	0	0.536364	0	0.58333333
3.92857143	0	Betaine aldehyde	Compound	0.25454545	0	102	NULL			C00576	C5H12NO	102.15488	249	1		6	C00576	9	1	0	0	0.511905	0	0
3.92857143	0	Choline phosphate	Compound	0.25454545	0	184	107-73-3			C00588	C5H15NO4P	184.150661	1014	2		6	C00588	9	2	0	1	0.511905	0	0
2.69090909	0	N-Acetyl-L-glutamate	Compound	0.37162162	0	189	1188-37-0			C00624	C7H11NO5	189.16594	7345	1		4	C00624	30	1	0	0	0.718182	0	0
2.65454545	0	5-Formiminotetrahydrofolate	Compound	0.37671233	1	472	NULL			C00664	C20H24N8O6	472.45456	530	3		4	C00664	12.33333333	2	1	0	0.724242	0	0.41111111
2.67272727	0	gamma-L-Glutamyl-L-cysteine	Compound	0.37414966	1	250	686-58-8			C00669	C8H14N2O5S	250.27216	123938	2		4	C00669	16	2	0	0	0.721212	0	0.53333333
1	1	sn-glycero-3-Phosphocholine	Input Compound	1	0	258	NULL	0.479	0.0044	C00670	C8H21NO6P	258.229201	439285	2	down	1	C00670	1	2	0	0	1	2	0
1.8	0	Lipoate	Compound	0.55555556	1	206	62-46-4			C00725	C8H14O2S2	206.32556	864	2		2	C00725	6	1	1	0	0.6	0	0.6
3.67857143	0	D-Sorbitol	Compound	0.27184466	0	182	50-70-4			C00794	C6H14O6	182.17176	5780	1		6	C00794	11	1	0	0	0.553571	0	0
3.29090909	0	L-Alanyl-tRNA	Compound	0.3038674	1	0	NULL			C00886	C13H22NO11P	0		2		5	C00886	7.5	2	0	0	0.618182	0	0.57692308
1.7	0	"1,3-Diaminopropane"	Compound	0.58823529	1	74	109-76-2			C00986	C3H10N2	74.1249	428	3		2	C00986	5.33333333	2	1	0	0.65	0	0.53333333
3.67857143	0	"alpha,alpha-Trehalose"	Compound	0.27184466	0	342	99-20-7			C01083	C12H22O11	342.29648	1143	1		6	C01083	11	1	0	0	0.553571	0	0
1	0	(R)-3-Hydroxybutanoate	Input Compound	1	0	104	NULL	1.31	0.0112	C01089	C4H8O3	104.10452	92135	1	up	1	C01089	1	1	0	0	1	0	0
4.32727273	0	trans-4-Hydroxy-L-proline	Compound	0.23109244	0	131	51-35-4			C01157	C5H9NO3	131.12986	5810	1		6	C01157	5	1	0	0	0.445455	0	0
2.69090909	0	L-Glutamate 5-semialdehyde	Compound	0.37162162	0	131	NULL			C01165	C5H9NO3	131.12986	193305	1		4	C01165	30	1	0	0	0.718182	0	0
3.21428571	0	1L-myo-Inositol 1-phosphate	Compound	0.31111111	0	260	NULL			C01177	C6H13O9P	260.135781	107737	1		4	C01177	8	1	0	0	0.630952	0	0
2.85714286	0	1-Phosphatidyl-D-myo-inositol	Compound	0.35	1	0	NULL			C01194	C11H17O13PR	0		2		4	C01194	6	2	0	0	0.690476	0	0.6
2.47272727	0	(R)-3-Amino-2-methylpropanoate	Compound	0.40441176	1	103	NULL			C01205	C4H9NO2	103.11976		6		4	C01205	12.2	4	2	1	0.754545	0	0.32105263
2.78571429	0	1-alpha-D-Galactosyl-myo-inositol	Compound	0.35897436	1	342	NULL			C01235	C12H22O11	342.29648	439451	2		4	C01235	6.5	2	0	0	0.702381	0	0.59090909
2.50909091	0.01526375	Cys-Gly	Compound	0.39855072	0.5	178	19246-18-5			C01419	C5H10N2O3S	178.2095	439498	4		4	C01419	11.75	2	2	0	0.748485	98	0.31756757
3.29090909	0	tRNA(Ala)	Compound	0.3038674	1	0	NULL			C01635	C10H17O10PR	0		2		5	C01635	7.5	1	1	0	0.618182	0	0.57692308
2.67272727	0	tRNA(Glu)	Compound	0.37414966	1	0	NULL			C01641	C15H21N5O10	0		2		4	C01641	16	1	1	0	0.721212	0	0.53333333
1.8	0	tRNA(Lys)	Compound	0.55555556	1	0	NULL			C01646	C10H17O10PR	0		2		2	C01646	6	1	1	0	0.6	0	0.6
4.30909091	0	tRNA(Pro)	Compound	0.23206751	1	0	NULL			C01649	C10H17O10PR	0		2		6	C01649	3.5	1	1	0	0.448485	0	0.7
3.89285714	0	Acylcholine	Compound	0.25688073	1	131	NULL			C01777	C6H13NO2R	0		2		6	C01777	5.5	2	0	0	0.517857	0	0.61111111
1.8	0	Choloyl-CoA	Compound	0.55555556	1	1157	NULL			C01794	C45H74N7O20	1158.090243	383	2		2	C01794	6	1	1	0	0.6	0	0.6
2.69090909	0	5-Oxoproline	Compound	0.37162162	0	129	98-79-3			C01879	C5H7NO3	129.11398	7405	1		4	C01879	30	1	0	0	0.718182	0	0
1.8	0	L-Lysyl-tRNA	Compound	0.55555556	1	145	NULL			C01931	C6H13N2O2R	0		2		2	C01931	6	2	0	0	0.6	0	0.6
3.92857143	0	Acetylcholine	Compound	0.25454545	0	146	51-84-3			C01996	C7H16NO2	146.20744	187	2		6	C01996	9	2	0	1	0.511905	0	0
2.67272727	0	R-S-Glutathione	Compound	0.37414966	1	0	NULL			C02320	C10H16N3O6S	0		2		4	C02320	16	2	0	0	0.721212	0	0.53333333
1.8	0	Taurolithocholate	Compound	0.55555556	1	483	516-90-5			C02592	C26H45NO5S	483.7042	439763	2		2	C02592	6	2	0	0	0.6	0	0.6
4.30909091	0	L-Prolyl-tRNA(Pro)	Compound	0.23206751	1	0	NULL			C02702	C15H24NO11P	0		2		6	C02702	3.5	2	0	0	0.448485	0	0.7
3.85714286	0	Phosphatidylserine	Compound	0.25925926	1	385	NULL			C02737	C8H12NO10PR	0	0	3		6	C02737	5.33333333	2	1	0	0.52381	0	0.59259259
2.67272727	0	L-Glutamyl-tRNA(Glu)	Compound	0.37414966	1	0	NULL			C02987	C20H28N6O13	0		2		4	C02987	16	2	0	0	0.721212	0	0.53333333
2.69090909	0	L-Glutamyl 5-phosphate	Compound	0.37162162	0	227	NULL			C03287	C5H10NO7P	227.109161	193475	1		4	C03287	30	1	0	0	0.718182	0	0
2.67272727	0	Tetrahydrofolyl-[Glu](n)	Compound	0.37414966	1	0	NULL			C03541	C24H30N8O9(	574.5432	442163	4		4	C03541	16	3	0	0	0.721212	0	0.53333333
3.21428571	0	myo-Inositol 4-phosphate	Compound	0.31111111	0	260	46495-39-0			C03546	C6H13O9P	260.135781	440043	1		4	C03546	8	1	0	0	0.630952	0	0
3.78181818	0	N-Acyl-O-acetylneuraminate	Compound	0.26442308	1	351	NULL			C03708	C12H18NO10R	0		2		6	C03708	7	2	0	0	0.536364	0	0.58333333
2.50909091	0.16835017	(S)-1-Pyrroline-5-carboxylate	Compound	0.39855072	0	113	NULL			C03912	C5H7NO2	113.11458		4		4	C03912	17.5	4	0	2	0.748485	510	0.5
1.8	0	3alpha-Hydroxy-5beta-cholanate	Compound	0.55555556	1	376	434-13-9			C03990	C24H40O3	376.5726	9903	2		2	C03990	6	1	1	0	0.6	0	0.6
3.21428571	0	1D-myo-Inositol 3-phosphate	Compound	0.31111111	0	260	NULL			C04006	C6H13O9P	260.135781	440194	1		4	C04006	8	1	0	0	0.630952	0	0
1.5	0	1-Acyl-sn-glycero-3-phosphocholine	Compound	0.66666667	0	285	NULL			C04230	C9H20NO7PR	0	5280634	1		2	C04230	2	1	0	0	0.75	0	0
1.5	0	2-Acyl-sn-glycero-3-phosphocholine	Compound	0.66666667	0	285	NULL			C04233	C9H20NO7PR	0		1		2	C04233	2	1	0	0	0.75	0	0
3.78181818	0	1-Organyl-2-lyso-sn-glycero-3-phosphocholine	Compound	0.26442308	1	257	NULL			C04317	C8H20NO6PR	0	0	2		6	C04317	7	1	1	0	0.536364	0	0.58333333
3.78181818	0	1-Alkyl-2-acetyl-sn-glycero-3-phosphocholine	Compound	0.26442308	1	299	NULL			C04598	C10H22NO7PR	0	2503	2		6	C04598	7	2	0	0	0.536364	0	0.58333333
1.8	0	Taurocholate	Compound	0.55555556	1	515	81-24-3			C05122	C26H45NO7S	515.703	440567	2		2	C05122	6	2	0	0	0.6	0	0.6
1.8	0	Chenodeoxycholoyl-CoA	Compound	0.55555556	1	1141	NULL			C05337	C45H74N7O19	1142.090843	11966205	2		2	C05337	6	1	1	0	0.6	0	0.6
3.03571429	0	Melibiose	Compound	0.32941176	1	342	585-99-9			C05402	C12H22O11	342.29648	440658	2		5	C05402	8	2	0	0	0.660714	0	0.61538462
1.8	0	Taurodeoxycholate	Compound	0.55555556	1	499	NULL			C05463	C26H45NO6S	499.7036	10594	2		2	C05463	6	2	0	0	0.6	0	0.6
1.8	0	Taurochenodeoxycholate	Compound	0.55555556	1	499	NULL			C05465	C26H45NO6S	499.7036	10591	2		2	C05465	6	2	0	0	0.6	0	0.6
1.8	0	N6-D-Biotinyl-L-lysine	Compound	0.55555556	1	372	NULL			C05552	C16H28N4O4S	372.48292	83814	2		2	C05552	6	2	0	0	0.6	0	0.6
2.67272727	0	R-S-Alanylglycine	Compound	0.37414966	1	0	NULL			C05729	C5H9N2O3SR	0		2		4	C05729	16	1	1	0	0.721212	0	0.53333333
1.9	0	5-Glutamyl-taurine	Compound	0.52631579	0	254	NULL			C05844	C7H14N2O6S	254.26086	0	1		2	C05844	10	1	0	0	0.55	0	0
3.8	0	Acetyl adenylate	Compound	0.26315789	0	389	NULL			C05993	C12H16N5O8P	389.257901	440867	1		6	C05993	12	1	0	0	0.533333	0	0
2.69090909	0	L-Glutamyl-tRNA(Gln)	Compound	0.37162162	0	0	NULL			C06112	C20H28N6O13	0		1		4	C06112	30	1	0	0	0.718182	0	0
2.67272727	0	Tetrahydrofolyl-[Glu](2)	Compound	0.37414966	1	574	NULL			C09332	C24H30N8O9	574.5432	442163	2		4	C09332	17	2	0	0	0.721212	0	0.56666667
3.78181818	0	4-Hydroxyphenyl acetate	Compound	0.26442308	1	152	3233-32-7			C13636	C8H8O3	152.14732		2		6	C13636	7	2	0	0	0.536364	0	0.58333333
3.27272727	0	gama-L-glutamyl-L-alanine	Compound	0.30555556	1	0	5875-41-2			CE0469	C8H13N2O5	0		2		5	CE0469	8.5	1	1	0	0.621212	0	0.60714286
3.30909091	0	N-acetyl-L-alanine	Compound	0.3021978	0	0	97-69-8			CE1554	C5H8NO3	0		2		5	CE1554	13	2	0	1	0.615152	0	0
1.8	0	deoxycholoyl-CoA	Compound	0.55555556	1	0	NULL			CE1589	C45H70N7O19	0		2		2	CE1589	6	1	1	0	0.6	0	0.6
1.8	0	lipoyllysine	Compound	0.55555556	1	0	20902-53-8			CE2102	C14H26N2O3S	0		2		2	CE2102	6	2	0	0	0.6	0	0.6
1.7	0	deoxyhypusine	Compound	0.58823529	1	0	NULL			CE5588	C10H25N3O2	0		3		2	CE5588	5.33333333	2	1	0	0.65	0	0.53333333
3.27272727	0	somatostatin	Compound	0.30555556	1	0	NULL			CE5782	C77H108N18O	0		3		5	CE5782	6.33333333	3	0	0	0.621212	0	0.48717949
3.27272727	0	Somatostatin fragment 3-14	Compound	0.30555556	1	0	NULL			CE5783	C72H100N16O	0		3		5	CE5783	6.33333333	1	2	0	0.621212	0	0.48717949

Table S6. Compounds and their network parameters related to the altered extracellular metabolites in endometrial stromal cells treated with HSP65 during decidualization

Average Shortest PathLength	Betweenness Centrality	canonical Name	Category	Closeness Centrality	Clustering Coefficient	Compound approximate MW	Compound casnum	Compound cid	Compound formula	Compound Media CvsHSP Fold change	Compound Media CvsHSP p value	Compound mw	Compound pubchemcid	Degree	direction	Eccentricity	name	Neighborhood Connectivity	Number Of Directed Edges	Number Of Undirected Edges	Partner Of Multi Edged Node Pairs	Radiality	Stress	Topological Coefficient
2.4	0	CoA	Compound	0.41666667	1	767	85-61-0	C00010	C21H36N7O16P3S			767.534123	6816	6		3	C00010	4.66666667	4	2	3	0.53333333	0	0.77777778
1.8	0	Peptide	Compound	0.55555556	0	0	NULL	C00012	C2H4NO2R(C2H2NOR)n			0		3		2	C00012	5	2	0	0	0.6	0	0
1.5	0	NH3	Compound	0.66666667	1	17	7664-41-7	C00014	H3N			17.03052	222	2		2	C00014	3	1	1	0	0.75	0	0.75
3.64285714	0	AMP	Compound	0.2745098	1	347	61-19-8	C00020	C10H14N5O7P			347.221221	6083	2		6	C00020	7.5	1	1	0	0.55952381	0	0.68181818
2	0.13043478	Pyruvate	Compound	0.5	0.4	88	127-17-3	C00022	C3H4O3			88.06206	1060	9		3	C00022	4.83333333	7	2	2	0.75	88	0.29166667
1.73333333	0.03174603	Acetyl-CoA	Compound	0.57692308	0.83333333	809	72-89-9	C00024	C23H38N7O17P3S			809.570803	6302	8		2	C00024	6.25	6	2	4	0.75555556	20	0.41666667
2.13043478	0	L-Glutamate	Compound	0.46938776	1	147	56-86-0	C00025	C5H9NO4			147.12926	33032	3		3	C00025	8.33333333	2	1	0	0.7173913	0	0.49019608
1.73913043	0.49802372	2-Oxoglutarate	Compound	0.575	0.4	146	328-50-7	C00026	C5H6O5			146.09814	51	6		2	C00026	6.16666667	4	2	0	0.81521739	288	0.26811594
2.71428571	0.48280423	D-Glucose	Input Compound	0.36842105	0.12727273	180	50-99-7	C00031	C6H12O6	0.463	3.00E-04	180.15588	5793	14	down	5	C00031	2.45454545	10	4	2	0.71428571	400	0.20661157
1.2	0.82857143	Acetate	Input Compound	0.83333333	0.09090909	60	64-19-7	C00033	C2H4O2	1.22	0.0072	60.05196	176	14	up	2	C00033	2.41666667	10	4	2	0.93333333	210	0.18055556
1.73333333	0.03174603	Oxaloacetate	Compound	0.57692308	0.83333333	132	328-42-7	C00036	C4H4O5			132.07156	970	8		2	C00036	6.25	5	3	3	0.75555556	20	0.41666667
2.56521739	0	Glycine	Compound	0.38983051	1	75	56-40-6	C00037	C2H5NO2			75.0666	750	3		4	C00037	6.33333333	1	2	0	0.60869565	0	0.48717949
1.69565217	0.60869565	L-Alanine	Input Compound	0.58974359	0.14102564	89	56-41-7	C00041	C3H7NO2	1.14	0.0038	89.09318	5950	16	up	3	C00041	3	11	5	2	0.82608696	330	0.18269231
2.08695652	0.45849802	Succinate	Input Compound	0.47916667	0.11111111	118	110-15-6	C00042	C4H6O4	0.638	1.00E-04	118.08804	1110	11	down	3	C00042	2.22222222	9	2	1	0.72826087	256	0.22222222
3.89285714	0	Carboxylate	Compound	0.25688073	1	44	NULL	C00060	CO2R			0		2		6	C00060	5.5	1	1	0	0.51785714	0	0.61111111
1.8125	0	L-Glutamine	Compound	0.55172414	1	146	56-85-9	C00064	C5H10N2O3			146.1445	5961	3		2	C00064	7.33333333	2	1	0	0.59375	0	0.45833333
3.85714286	0	L-Serine	Compound	0.25925926	1	105	56-45-1	C00065	C3H7NO3			105.09258	5951	3		6	C00065	5.33333333	2	1	0	0.52380952	0	0.59259259
1	0.84166667	L-Phenylalanine	Input Compound	1	0.14166667	165	63-91-2	C00079	C9H11NO2	0.869	0.0039	165.18914	6140	23	down	1	C00079	3.125	14	9	5	1	206	0.21428571
1.75	0.00277778	L-Tyrosine	Compound	0.57142857	0.83333333	181	60-18-4	C00082	C9H11NO3			181.18854	6057	7		2	C00082	6.5	5	2	2	0.625	2	0.40625
2.13333333	0	Acetaldehyde	Compound	0.46875	0	44	75-07-0	C00084	C2H4O			44.05256	177	2		3	C00084	12	2	0	1	0.62222222	0	0
3.64285714	0	Sucrose	Compound	0.2745098	1	342	57-50-1	C00089	C12H22O11			342.29648	5988	2		6	C00089	6.5	2	0	0	0.55952381	0	0.59090909
3.04347826	0	Succinyl-CoA	Compound	0.32857143	0	867	604-98-8	C00091	C25H40N7O19P3S			867.606883	439161	3		4	C00091	9	3	0	1	0.48913043	0	0
3.57142857	0.0026455	D-Glucose 6-phosphate	Compound	0.28	0.66666667	260	56-73-5	C00092	C6H13O9P			260.135781	208	6		6	C00092	4.75	4	2	1	0.57142857	4	0.43181818
3.64285714	0	D-Fructose	Compound	0.2745098	1	180	57-48-7	C00095	C6H12O6			180.15588		2		6	C00095	6.5	1	1	0	0.55952381	0	0.59090909
2.96428571	0.38095238	Choline	Input Compound	0.3373494	0.13888889	104	62-49-7	C00114	C5H14NO	0.583	0.0038	104.17076	305	12	down	5	C00114	2.33333333	9	3	3	0.67261905	328	0.25
3.04347826	0	Fumarate	Compound	0.32857143	0	116	110-17-8	C00122	C4H4O4			116.07216	723	1		4	C00122	9	1	0	0	0.48913043	0	0
1.6875	0.00555556	L-Leucine	Compound	0.59259259	0.8	131	61-90-5	C00123	C6H13NO2			131.17292	6106	7		2	C00123	6.4	2	5	2	0.65625	4	0.4
2.39285714	0.49603175	D-Galactose	Compound	0.41791045	0.3	180	59-23-4	C00124	C6H12O6			180.15588	439357	6		4	C00124	5	3	3	1	0.76785714	420	0.26315789
3.60714286	0	dATP	Compound	0.27722772	1	491	1927-31-7	C00131	C10H16N5O12P3			491.181623	15993	3		6	C00131	6	2	1	0	0.56547619	0	0.54545455
2.25	0.64814815	myo-Inositol	Input Compound	0.44444444	0.07142857	180	87-89-8	C00137	C6H12O6	0.673	1.00E-04	180.15588	892	8	down	3	C00137	2.125	6	2	0	0.79166667	558	0.18055556
1	0.9	L-Proline	Input Compound	1	0.1	115	147-85-3	C00148	C5H9NO2	0.64	1.00E-04	115.13046	145742	6	down	1	C00148	1.4	5	1	1	1	18	0.4
3.14285714	0.05291005	Phosphatidylcholine	Compound	0.31818182	0.66666667	2049	8002-43-5	C00157	C10H18NO8PR2			0	452110	5		5	C00157	4.75	4	1	1	0.64285714	80	0.43181818
1.6	0.28888889	Citrate	Input Compound	0.625	0.33333333	192	77-92-9	C00158	C6H8O7	0.31	6.00E-04	192.12352	311	10	down	2	C00158	4.16666667	8	2	3	0.8	74	0.29487179
3.04347826	0	Acetoacetate	Compound	0.32857143	0	102	541-50-4	C00164	C4H6O3			102.08864	96	1		4	C00164	9	1	0	0	0.48913043	0	0
1.8125	0	Phenylpyruvate	Compound	0.55172414	1	164	156-06-9	C00166	C9H8O3			164.15802	997	5		2	C00166	7.33333333	4	1	1	0.59375	0	0.45833333
2.95652174	0	(S)-Lactate	Input Compound	0.33823529	0	90	79-33-4	C00186	C3H6O3	0.537	0.0081	90.07794	107689	2	down	4	C00186	6	2	0	1	0.51086957	0	0
3.21428571	0	D-Glucuronate	Compound	0.31111111	0	194	12/3/1956	C00191	C6H10O7			194.1394	444791	1		4	C00191	8	1	0	0	0.63095238	0	0
3.60714286	0	dADP	Compound	0.27722772	1	411	NULL	C00206	C10H15N5O9P2			411.201722	188966	3		6	C00206	6	2	1	0	0.56547619	0	0.54545455
2.13333333	0	Acetyl phosphate	Compound	0.46875	0	140	NULL	C00227	C2H5O5P			140.031861	186	1		3	C00227	12	1	0	0	0.62222222	0	0
3.04347826	0	Succinate semialdehyde	Compound	0.32857143	0	102	692-29-5	C00232	C4H6O3			102.08864	1112	1		4	C00232	9	1	0	0	0.48913043	0	0
3.03571429	0	Lactose	Compound	0.32941176	1	342	63-42-3	C00243	C12H22O11			342.29648	84571	2		5	C00243	8	2	0	0	0.66071429	0	0.61538462
1	0.91111111	Taurine	Input Compound	1	0.08888889	125	107-35-7	C00245	C2H7NO3S	0.842	0.0234	125.14688	1123	10	down	1	C00245	1.8	6	4	0	1	82	0.2
1.8125	0	Dihydrobiopterin	Compound	0.55172414	1	239	NULL	C00268	C9H13N5O3			239.23122		3		2	C00268	8	2	1	0	0.59375	0	0.5
2.06666667	0	N-Acetylneuraminate	Compound	0.48387097	1	309	131-48-6	C00270	C11H19NO9			309.26986	439197	2		3	C00270	7	1	1	0	0.64444444	0	0.58333333
1.75	0.00277778	Tetrahydrobiopterin	Compound	0.57142857	0.83333333	241	17528-72-2	C00272	C9H15N5O3			241.2471	1125	6		2	C00272	6.5	4	2	2	0.625	2	0.40625
2.53333333	0	Isocitrate	Compound	0.39473684	0	192	320-77-4	C00311	C6H8O7			192.12352	1198	1		3	C00311	6	1	0	0	0.48888889	0	0
2.52173913	0	2-Methyl-3-oxopropanoate	Compound	0.39655172	1	102	NULL	C00349	C4H6O3			102.08864	296	3		4	C00349	7.33333333	2	1	0	0.61956522	0	0.52380952
1	0.66666667	L-Isoleucine	Input Compound	1	0.33333333	131	73-32-5	C00407	C6H13NO2	0.564	1.00E-04	131.17292	791	5	down	1	C00407	2	4	1	1	1	8	0.5
2.53571429	0.45238095	Phosphatidate	Compound	0.3943662	0.33333333	596	NULL	C00416	C5H7O8PR2			0	5873088	4		4	C00416	5.75	2	2	0	0.74404762	418	0.33823529
2.53333333	0	cis-Aconitate	Compound	0.39473684	0	174	585-84-2	C00417	C6H6O6			174.10824	309	1		3	C00417	6	1	0	0	0.48888889	0	0
1.9	0	L-Cysteate	Compound	0.52631579	0	169	13100-82-8	C00506	C3H7NO5S			169.15638	25701	1		2	C00506	10	1	0	0	0.55	0	0
2.06666667	0	Hydroquinone	Compound	0.48387097	1	110	123-31-9	C00530	C6H6O2			110.11064	785	2		3	C00530	7	1	1	0	0.64444444	0	0.58333333
3.92857143	0	Betaine aldehyde	Compound	0.25454545	0	102	NULL	C00576	C5H12NO			102.15488	249	1		6	C00576	9	1	0	0	0.51190476	0	0
3.92857143	0	Choline phosphate	Compound	0.25454545	0	184	107-73-3	C00588	C5H15NO4P			184.150661	1014	2		6	C00588	9	2	0	1	0.51190476	0	0
3.67857143	0	D-Sorbitol	Compound	0.27184466	0	182	50-70-4	C00794	C6H14O6			182.17176	5780	1		6	C00794	11	1	0	0	0.55357143	0	0
2.60869565	0	L-Alanyl-tRNA	Compound	0.38333333	1	0	NULL	C00886	C13H22NO11PR2(C5H8O6PR)n			0		2		4	C00886	7.5	2	0	0	0.59782609	0	0.57692308
1.8125	0	2-Oxoglutaramate	Compound	0.55172414	1	145	NULL	C00940	C5H7NO4			145.11338	48	3		2	C00940	7.33333333	2	1	0	0.59375	0	0.45833333
3.67857143	0	"alpha,alpha-Trehalose"	Compound	0.27184466	0	342	99-20-7	C01083	C12H22O11			342.29648	1143	1		6	C01083	11	1	0	0	0.55357143	0	0
1.8	0	trans-4-Hydroxy-L-proline	Compound	0.55555556	0	131	51-35-4	C01157	C5H9NO3			131.12986	5810	1		2	C01157	5	1	0	0	0.6	0	0
3.21428571	0	1L-myo-Inositol 1-phosphate	Compound	0.31111111	0	260	NULL	C01177	C6H13O9P			260.135781	107737	1		4	C01177	8	1	0	0	0.63095238	0	0
2.85714286	0	1-Phosphatidyl-D-myo-inositol	Compound	0.35	1	0	NULL	C01194	C11H17O13PR2			0		2		4	C01194	6	2	0	0	0.69047619	0	0.6
2.52173913	0	(R)-3-Amino-2-methylpropanoate	Compound	0.39655172	1	103	NULL	C01205	C4H9NO2			103.11976		3		4	C01205	7.33333333	2	1	0	0.61956522	0	0.52380952
2.78571429	0	1-alpha-D-Galactosyl-myo-inositol	Compound	0.35897436	1	342	NULL	C01235	C12H22O11			342.29648	439451	2		4	C01235	6.5	2	0	0	0.70238095	0	0.59090909
2.60869565	0	Cys-Gly	Compound	0.38333333	1	178	19246-18-5	C01419	C5H10N2O3S			178.2095	439498	2		4	C01419	7.5	1	1	0	0.59782609	0	0.57692308
2.60869565	0	tRNA(Ala)	Compound	0.38333333	1	0	NULL	C01635	C10H17O10PR2(C5H8O6PR)n			0		2		4	C01635	7.5	1	1	0	0.59782609	0	0.57692308
1.5	0	tRNA(Ile)	Compound	0.66666667	1	0	NULL	C01644	C15H21N5O10PR(C5H8O6PR)n			0		2		2	C01644	3	1	1	0	0.75	0	0.75
1.875	0	tRNA(Phe)	Compound	0.53333333	1	0	NULL	C01648	C10H17O10PR2(C5H8O6PR)n			0		2		2	C01648	9	1	1	0	0.5625	0	0.5625
1.6	0	tRNA(Pro)	Compound	0.625	1	0	NULL	C01649	C10H17O10PR2(C5H8O6PR)n			0		2		2	C01649	3.5	1	1	0	0.7	0	0.7
3.89285714	0	Acylcholine	Compound	0.25688073	1	131	NULL	C01777	C6H13NO2R			0		2		6	C01777	5.5	2	0	0	0.51785714	0	0.61111111
1.8	0	Choloyl-CoA	Compound	0.55555556	1	1157	NULL	C01794	C45H74N7O20P3S			1158.090243	383	2		2	C01794	6	1	1	0	0.6	0	0.6
3.92857143	0	Acetylcholine	Compound	0.25454545	0	146	51-84-3	C01996	C7H16NO2			146.20744	187	2		6	C01996	9	2	0	1	0.51190476	0	0
1.9375	0	2-Phenylacetamide	Compound	0.51612903	0	135	103-81-1	C02505	C8H9NO			135.16316	7680	1		2	C02505	16	1	0	0	0.53125	0	0
1.8	0	Taurolithocholate	Compound	0.55555556	1	483	516-90-5	C02592	C26H45NO5S			483.7042	439763	2		2	C02592	6	2	0	0	0.6	0	0.6
1.6	0	L-Prolyl-tRNA(Pro)	Compound	0.625	1	0	NULL	C02702	C15H24NO11PR2(C5H8O6PR)n			0		2		2	C02702	3.5	2	0	0	0.7	0	0.7
3.85714286	0	Phosphatidylserine	Compound	0.25925926	1	385	NULL	C02737	C8H12NO10PR2			0	0	3		6	C02737	5.33333333	2	1	0	0.52380952	0	0.59259259
2.34782609	0	Peptide L-aspartate	Compound	0.42592593	1	0	NULL	C02871	C6H9N2O5R(C2H2NOR)n			0		3		3	C02871	6	2	1	0	0.66304348	0	0.5
1.5	0	L-Isoleucyl-tRNA(Ile)	Compound	0.66666667	1	0	NULL	C03127	C21H32N6O11PR(C5H8O6PR)n			0		2		2	C03127	3	2	0	0	0.75	0	0.75
1.5	0	3-Methyl-2-oxopentanoate	Compound	0.66666667	1	130	NULL	C03465	C6H10O3			130.1418		3		2	C03465	3	2	1	1	0.75	0	0.75
1.875	0	L-Phenylalanyl-tRNA(Phe)	Compound	0.53333333	1	0	NULL	C03511	C19H26NO11PR2(C5H8O6PR)n			0		2		2	C03511	9	2	0	0	0.5625	0	0.5625
3.21428571	0	myo-Inositol 4-phosphate	Compound	0.31111111	0	260	46495-39-0	C03546	C6H13O9P			260.135781	440043	1		4	C03546	8	1	0	0	0.63095238	0	0
2.06666667	0	N-Acyl-O-acetylneuraminate	Compound	0.48387097	1	351	NULL	C03708	C12H18NO10R			0		2		3	C03708	7	2	0	0	0.64444444	0	0.58333333
1.8	0	(S)-1-Pyrroline-5-carboxylate	Compound	0.55555556	0	113	NULL	C03912	C5H7NO2			113.11458		2		2	C03912	5	2	0	1	0.6	0	0
2.34782609	0	Peptide 3-hydroxy-L-aspartate	Compound	0.42592593	1	0	NULL	C03957	C6H9N2O6R(C2H2NOR)n			0		3		3	C03957	6	2	1	0	0.66304348	0	0.5
1.8	0	3alpha-Hydroxy-5beta-cholanate	Compound	0.55555556	1	376	434-13-9	C03990	C24H40O3			376.5726	9903	2		2	C03990	6	1	1	0	0.6	0	0.6
3.21428571	0	1D-myo-Inositol 3-phosphate	Compound	0.31111111	0	260	NULL	C04006	C6H13O9P			260.135781	440194	1		4	C04006	8	1	0	0	0.63095238	0	0
2.06666667	0	1-Organyl-2-lyso-sn-glycero-3-phosphocholine	Compound	0.48387097	1	257	NULL	C04317	C8H20NO6PR			0	0	2		3	C04317	7	1	1	0	0.64444444	0	0.58333333
2.06666667	0	1-Alkyl-2-acetyl-sn-glycero-3-phosphocholine	Compound	0.48387097	1	299	NULL	C04598	C10H22NO7PR			0	2503	2		3	C04598	7	2	0	0	0.64444444	0	0.58333333
1.8	0	Taurocholate	Compound	0.55555556	1	515	81-24-3	C05122	C26H45NO7S			515.703	440567	2		2	C05122	6	2	0	0	0.6	0	0.6
1.9375	0	Phenethylamine	Compound	0.51612903	0	121	64-04-0	C05332	C8H11N			121.17964	1001	1		2	C05332	16	1	0	0	0.53125	0	0
1.8	0	Chenodeoxycholoyl-CoA	Compound	0.55555556	1	1141	NULL	C05337	C45H74N7O19P3S			1142.090843	11966205	2		2	C05337	6	1	1	0	0.6	0	0.6
3.03571429	0	Melibiose	Compound	0.32941176	1	342	585-99-9	C05402	C12H22O11			342.29648	440658	2		5	C05402	8	2	0	0	0.66071429	0	0.61538462
1.8	0	Taurodeoxycholate	Compound	0.55555556	1	499	NULL	C05463	C26H45NO6S			499.7036	10594	2		2	C05463	6	2	0	0	0.6	0	0.6
1.8	0	Taurochenodeoxycholate	Compound	0.55555556	1	499	NULL	C05465	C26H45NO6S			499.7036	10591	2		2	C05465	6	2	0	0	0.6	0	0.6
1.9	0	5-Glutamyl-taurine	Compound	0.52631579	0	254	NULL	C05844	C7H14N2O6S			254.26086	0	1		2	C05844	10	1	0	0	0.55	0	0
2.13333333	0	Acetyl adenylate	Compound	0.46875	0	389	NULL	C05993	C12H16N5O8P			389.257901	440867	1		3	C05993	12	1	0	0	0.62222222	0	0
2.06666667	0	4-Hydroxyphenyl acetate	Compound	0.48387097	1	152	3233-32-7	C13636	C8H8O3			152.14732		2		3	C13636	7	2	0	0	0.64444444	0	0.58333333
2.60869565	0	gama-L-glutamyl-L-alanine	Compound	0.38333333	1	0	5875-41-2	CE0469	C8H13N2O5			0		2		4	CE0469	7.5	1	1	0	0.59782609	0	0.57692308
2.65217391	0	N-acetyl-L-alanine	Compound	0.37704918	0	0	97-69-8	CE1554	C5H8NO3			0		2		4	CE1554	13	2	0	1	0.58695652	0	0
1.8	0	deoxycholoyl-CoA	Compound	0.55555556	1	0	NULL	CE1589	C45H70N7O19P3S			0		2		2	CE1589	6	1	1	0	0.6	0	0.6
1.8125	0	tetrahydrobiopterin-4a-carbinolamine	Compound	0.55172414	1	0	NULL	CE1951	C9H16N5O4			0		3		2	CE1951	8	2	1	0	0.59375	0	0.5
3	0	3(S)-phytanoyl-CoA	Compound	0.33333333	1	0	NULL	CE5122	C41H70N7O17P3S			0		2		4	CE5122	5.5	2	0	0	0.5	0	0.61111111
3	0	3(S)-2-hydroxyphytanoyl-CoA	Compound	0.33333333	1	0	NULL	CE5123	C41H70N7O18P3S			0		2		4	CE5123	5.5	1	1	0	0.5	0	0.61111111
2.56521739	0	somatostatin	Compound	0.38983051	1	0	NULL	CE5782	C77H108N18O19S2			0		3		4	CE5782	6.33333333	3	0	0	0.60869565	0	0.48717949
2.56521739	0	Somatostatin fragment 3-14	Compound	0.38983051	1	0	NULL	CE5783	C72H100N16O17S2			0		3		4	CE5783	6.33333333	1	2	0	0.60869565	0	0.48717949
1.6875	0.00555556	kinetensin	Compound	0.59259259	0.8	0	NULL	CE5786	C56H87N17O11			0		7		2	CE5786	6.4	7	0	2	0.65625	4	0.4
1.75	0	kinetensin 1-3	Compound	0.57142857	1	0	NULL	CE5787	C15H31N6O4			0		4		2	CE5787	7.5	1	3	0	0.625	0	0.46875
1.8125	0	kinetensin 1-7	Compound	0.55172414	1	0	NULL	CE5788	C41H67N15O9			0		3		2	CE5788	8.66666667	1	2	0	0.59375	0	0.54166667
1.75	0	kinetensin 4-7	Compound	0.57142857	1	0	NULL	CE5790	C26H38N9O6			0		4		2	CE5790	7.5	1	3	0	0.625	0	0.46875

SUPPLEMENTARY FIGURE LEGENDS

Figure S1: Two dimensional scatter score of plot of PCA (**A**) and PLS-DA (**B**) obtained by comparing ND, DEC and DEC+HSP65.

Figure S2: Representative 700 MHz NMR spectra (δ 0.0−9.0) of hESC (**A**) and culture media (**B**) during in vitro decidualization. Metabolite assignments are indicated as follows: 1.TSP, 2.Lipid, 3.L-Isoleucine, 4.L-Leucine, 5.L-Valine, 6.3-Hydroxybutyrate, 7.Lactate, 8.L-Alanine, 9. L-Lysine, 10.Acetate, 11.L-Proline, 12.L-Glutamine, 13.D-Glutamate, 14.Succinate, 15.Pyruvate, 16.Citrate, 17.Choline, 18.Glycerophosphocholine, 19.Taurine, 20.Myoinositol, 21.Glycine, 22.D-Glucose, 23.L-Histidine, 24.L-Threonine, 25-L-Tyrosine, 26.L-Phenylalanine, 27.Formate, 28.1-Methyl histidine, 29.Oxypurinol.

Figure S3: Scatter plot of PCA [R2X = 70.1% and Q2 = 0.455] (**A**), PLS-DA of ND and d3, d6 and d9 metabolomic profiles of DEC and DEC+HSP65 [R2X = 62.5%, R2Y = 0.616 and Q2 = 0.411] (**B**).

Figure S4: The heatmap shows the Pearson's method based correlation between extracelluar metabolites in ND, DEC and DEC+HSP65 (**A-C**).

Figure S5: The statistical correlation between intracellular and extracelluar metabolites in ND, DEC and DEC+HSP65 are represented (**A-C**).

Figure S6: HSP65 mediated metabolic pathways and networks. (**A**) Metabolic pathway analysis shows the potential pathways dysregulated in HSP65 treated hESC during decidualization. Node size and color indicate the degree of importance. Large red node represents the highest level of changes in the HSP65 treated cells. Moderate, slight and zero importance pathways are represented by orange, yellow and white nodes, respectively. Pathway impact score indicates

the altered metabolic pathways. (**B**) HSP65 mediated metabolic network analyzed by Cytoscape based Network Analyzer plug-in represents the global and node network parameters. (**C**) The network follows the power law distribution, represents the specificity of HSP65 induced network.

Figure S7: HSP65 centered pathways and networks based on identified metabolites in culture media of hESC. (**A**) Altered Metabolic pathways associated with dysregulated metabolites in culture media of HSP65 treated decidualized cells. (**B**) Network parameters of HSP65 induced network. (**C**) Power law distribution of the network indicating the specificity of the generated modules.

Figure S8: HSP65 centered metabolic networks. (**A**) Compound network map of perturbed intracellular metabolites is indicating the potentially altered metabolic pathways in HSP65 treated decidualized cells. (**B**) Metabolites with high degree indicate majorly contributing metabolites to the altered pathways. Input compound-Red hexagon with rim colored in green Pink hexagon - Related compounds.

Figure S9: (**A**) Compound network modules represent potentially important pathways based on identified metabolites in culture media of hESC under HSP65 stimuli. (**B**) Metabolites with high degree (>8) in culture media of hESC treated with HSP65 are indicated.

Figure S10: Markov clustering of metabolic networks for each dysregulated extracellular metabolites in HSP65 treated decidualized cells. Input compound-red hexagon with green rim and Input metabolites related compounds-pink hexagon.

Figure S1

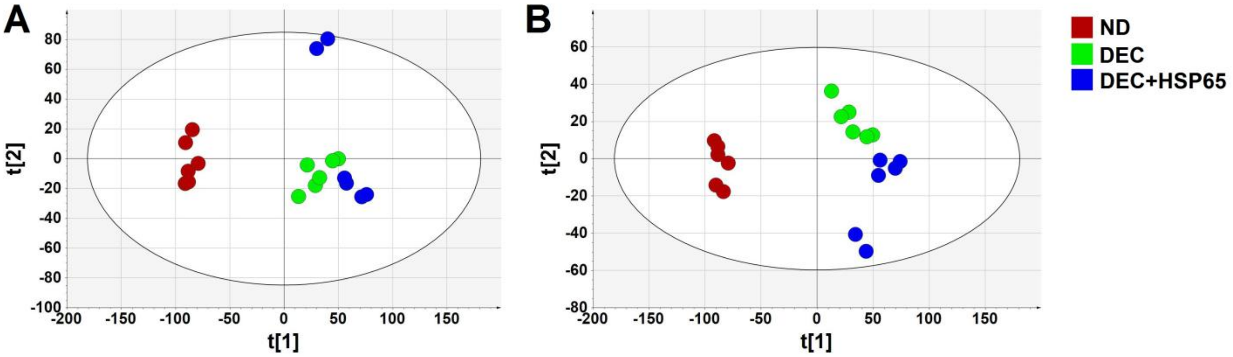

Figure S2

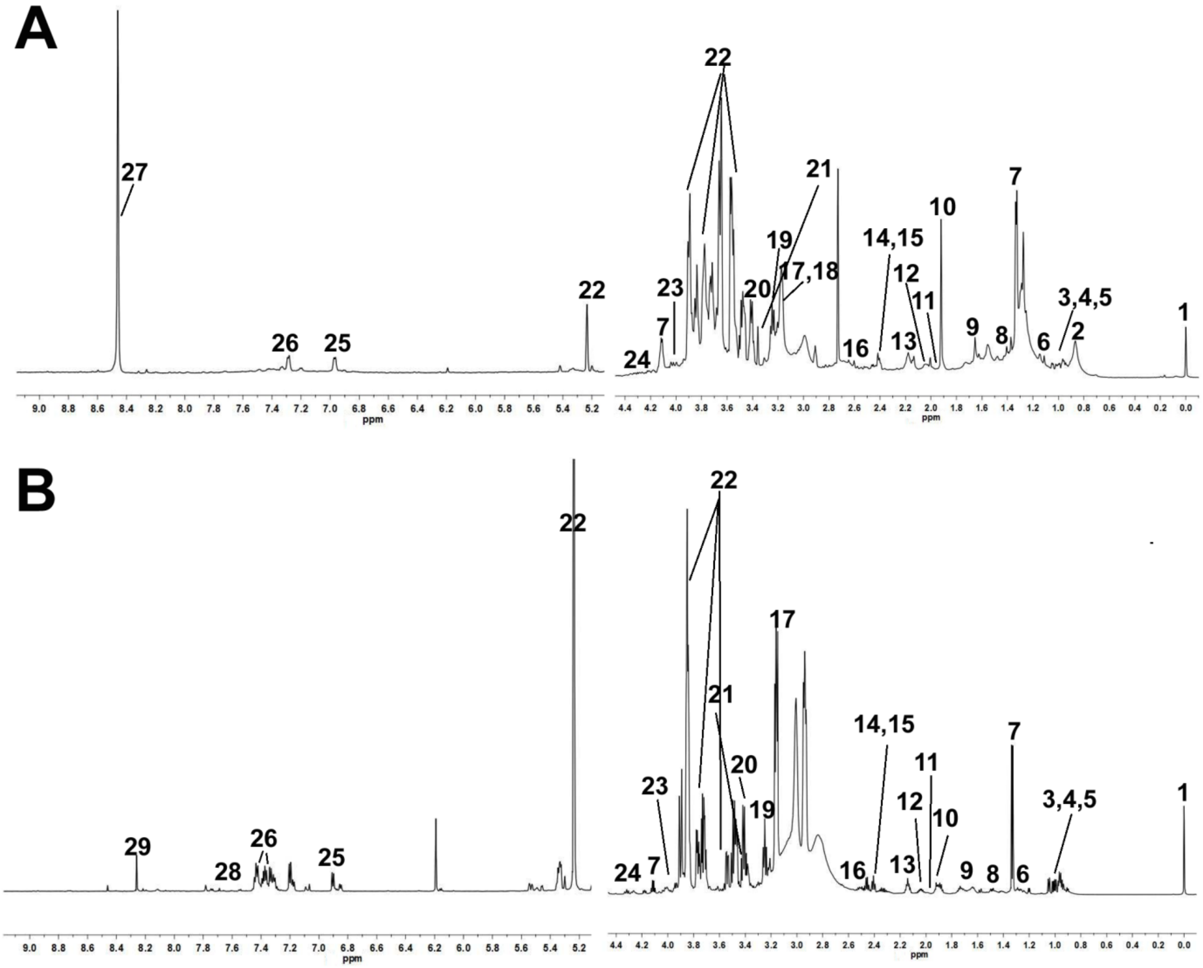

A
27
26
25
22
22
24
7
23
20
19
21
17,18
16
14,15
13
12
11
10
9
8
7
6
3,4,5
2
1
ppm
B
29
28
26
25
22
22
24
7
23
21
20
19
17
16
14,15
13
12
11
10
9
8
7
6
3,4,5
1
ppm

Figure S3

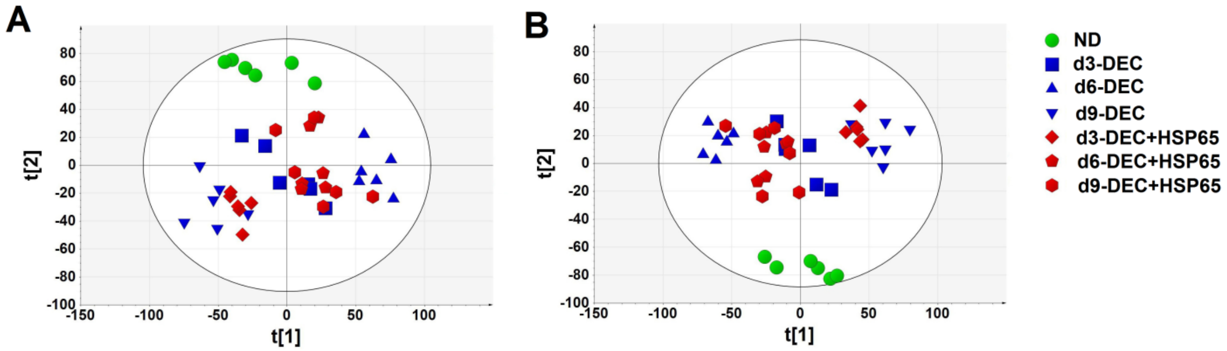
A
B
t[2]
t[1]
80
60
40
20
0
-20
-40
-60
-80
-100
-150
-100
-50
0
50
100
ND
d3-DEC
d6-DEC
d9-DEC
d3-DEC+HSP65
d6-DEC+HSP65
d9-DEC+HSP65

Figure S4

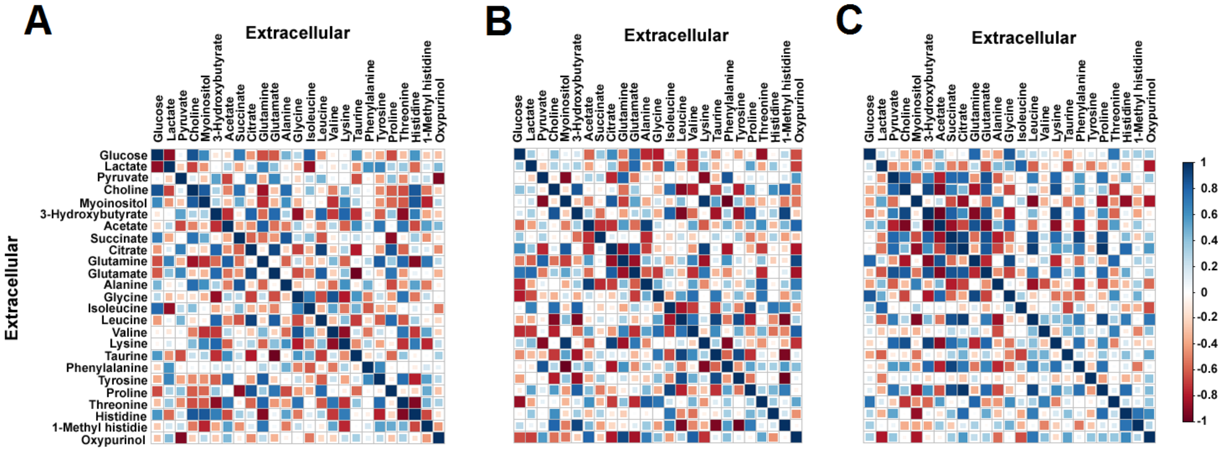
A
B
C
Extracellular
Extracellular
Glucose
Lactate
Pyruvate
Choline
Myoinositol
3-Hydroxybutyrate
Acetate
Succinate
Citrate
Glutamine
Glutamate
Alanine
Glycine
Isoleucine
Leucine
Valine
Lysine
Taurine
Phenylalanine
Tyrosine
Proline
Threonine
Histidine
1-Methyl histidine
1-Methyl histidie
Oxypurinol
1
0.8
0.6
0.4
0.2
0
-0.2
-0.4
-0.6
-0.8
-1

Figure S5

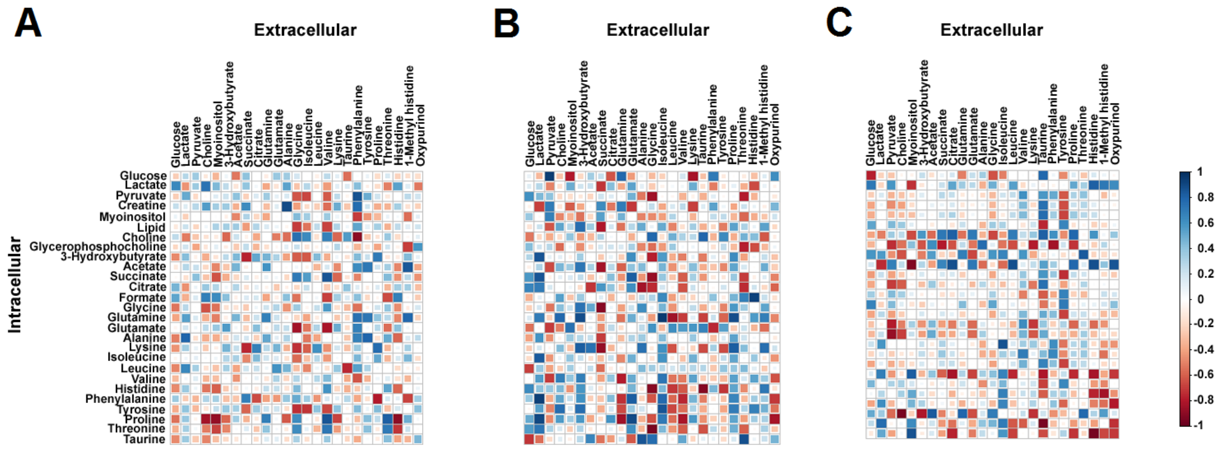
A
B
C
Extracellular
Intracellular
Glucose
Lactate
Pyruvate
Creatine
Myoinositol
Lipid
Choline
Glycerophosphocholine
3-Hydroxybutyrate
Acetate
Succinate
Citrate
Formate
Glycine
Glutamine
Glutamate
Alanine
Lysine
Isoleucine
Leucine
Valine
Histidine
Phenylalanine
Tyrosine
Proline
Threonine
Taurine
1-Methyl histidine
Oxypurinol
1
0.8
0.6
0.4
0.2
0
-0.2
-0.4
-0.6
-0.8
-1

Figure S6

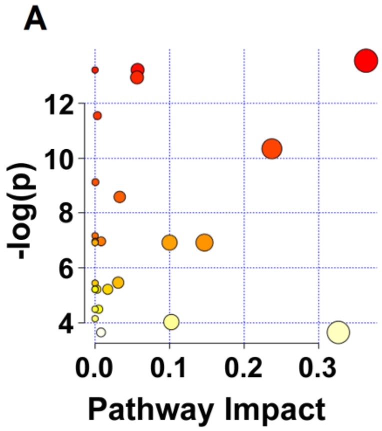

B

Network Parameters	
Number of nodes	112
Clustering coefficient	0.602
Connected components	6
Network diameter	6
Network radius	1
Network centralization	0.248
Characteristic path length	3.025
Average number of neighbors	2.964
Network density	0.027
Network heterogeneity	1.173

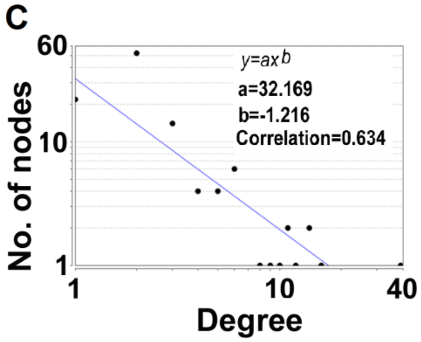

Figure S7

A

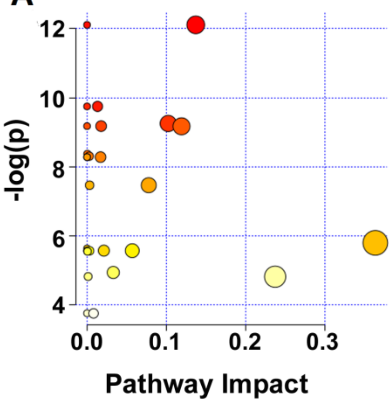

B

Network Parameters	
Number of nodes	108
Clustering coefficient	0.624
Connected components	7
Network diameter	6
Network radius	1
Network centralization	0.124
Characteristic path length	2.632
Average number of neighbors	3.0
Network density	0.028
Network heterogeneity	0.883

C

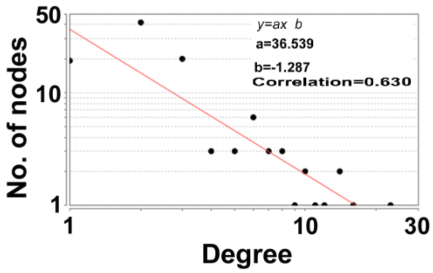

Figure S8

A

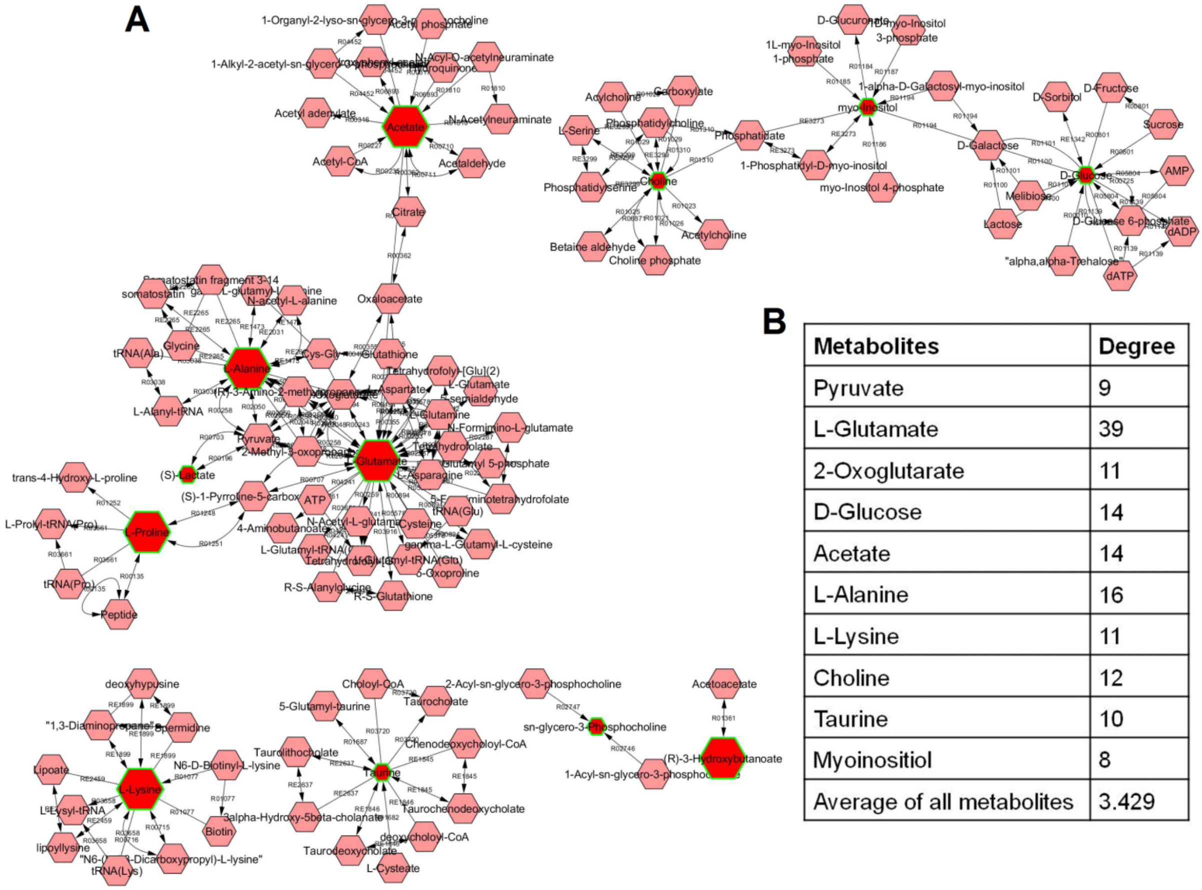

B

Metabolites	Degree
Pyruvate	9
L-Glutamate	39
2-Oxoglutarate	11
D-Glucose	14
Acetate	14
L-Alanine	16
L-Lysine	11
Choline	12
Taurine	10
Myoinositiol	8
Average of all metabolites	3.429

Figure S9

A

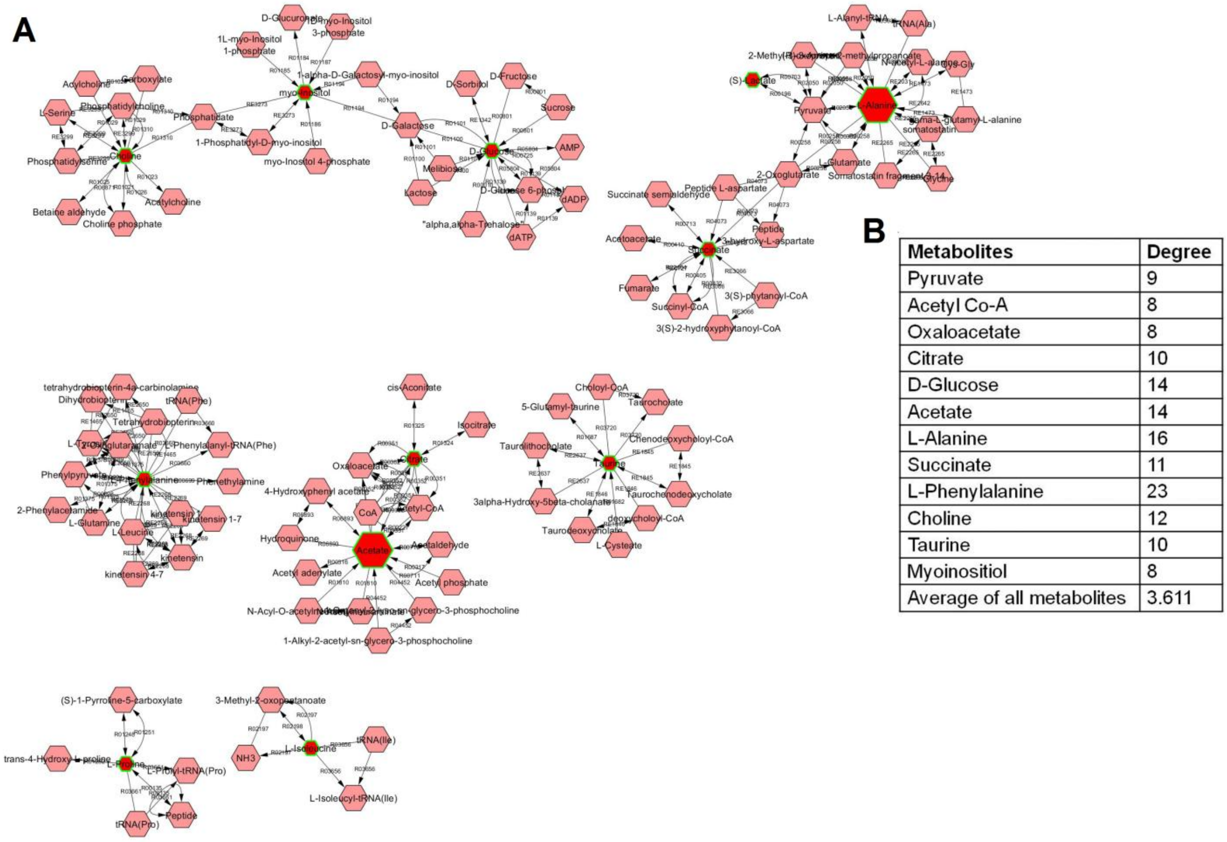

B

Metabolites	Degree
Pyruvate	9
Acetyl Co-A	8
Oxaloacetate	8
Citrate	10
D-Glucose	14
Acetate	14
L-Alanine	16
Succinate	11
L-Phenylalanine	23
Choline	12
Taurine	10
Myoinositiol	8
Average of all metabolites	3.611

Figure S10

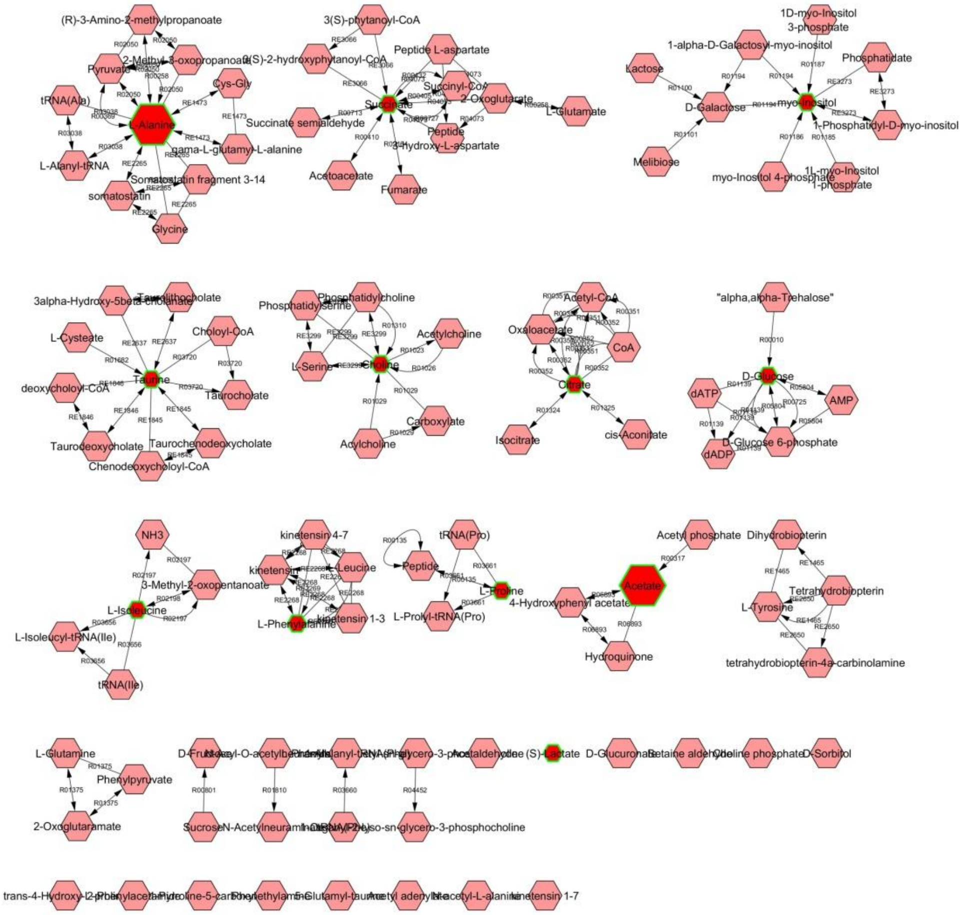

(R)-3-Amino-2-methylpropanoate
Pyruvate
Cys-Gly
tRNA(Ala)
L-Alanine
L-Alanyl-tRNA
gama-L-glutamyl-L-alanine
somatostatin
Glycine
3(S)-phytanoyl-CoA
Peptide L-aspartate
Succinyl-CoA
2-Oxoglutarate
L-Glutamate
Succinate
Succinate semialdehyde
Peptide
3-Hydroxy-L-aspartate
Acetoacetate
Fumarate
1D-myo-Inositol
3-phosphate
1-alpha-D-Galactosyl-myo-inositol
Lactose
Phosphatidate
D-Galactose
1-Phosphatidyl-D-myo-inositol
Melibiose
myo-Inositol 4-phosphate
1L-myo-Inositol
1-phosphate
L-Cysteate
Choloyl-CoA
Taurine
deoxycholoyl-CoA
Taurocholate
Taurodeoxycholate
Taurochenodeoxycholate
Chenodeoxycholoyl-CoA
Phosphatidylserine
Phosphatidylcholine
Acetylcholine
L-Serine
Choline
Carboxylate
Acylcholine
Acetyl-CoA
Oxaloacetate
CoA
Citrate
Isocitrate
cis-Aconitate
"alpha,alpha-Trehalose"
D-Glucose
dATP
AMP
dADP
D-Glucose 6-phosphate
NH3
3-Methyl-2-oxopentanoate
L-Isoleucine
L-Isoleucyl-tRNA(Ile)
tRNA(Ile)
kinetensin 4-7
L-Leucine
L-Phenylalanine
tRNA(Pro)
L-Proline
L-Prolyl-tRNA(Pro)
Acetyl phosphate
Acetate
4-Hydroxyphenyl acetate
Hydroquinone
Dihydrobiopterin
Tetrahydrobiopterin
L-Tyrosine
tetrahydrobiopterin-4a-carbinolamine
L-Glutamine
Phenylpyruvate
2-Oxoglutaramate
Sucrose
D-Glucuronate
Betaine aldehyde
D-Sorbitol
kinetensin 1-7
